# Altitude and life‐history shape the evolution of *Heliconius* wings

**DOI:** 10.1111/evo.13865

**Published:** 2019-11-06

**Authors:** Gabriela Montejo‐Kovacevich, Jennifer E. Smith, Joana I. Meier, Caroline N. Bacquet, Eva Whiltshire‐Romero, Nicola J. Nadeau, Chris D. Jiggins

**Affiliations:** ^1^ Department of Zoology University of Cambridge Cambridge CB2 3EJ UK; ^2^ Animal and Plant Sciences University of Sheffield Sheffield S10 2TN UK; ^3^ St John's College University of Cambridge Cambridge CB2 1TP; ^4^ Universidad Regional Amazónica Ikiam Tena Ecuador

**Keywords:** Altitude, *Heliconius*, Lepidoptera, phenotypic divergence, sexual dimorphism, wing morphology

## Abstract

Phenotypic divergence between closely related species has long interested biologists. Taxa that inhabit a range of environments and have diverse natural histories can help understand how selection drives phenotypic divergence. In butterflies, wing color patterns have been extensively studied but diversity in wing shape and size is less well understood. Here, we assess the relative importance of phylogenetic relatedness, natural history, and habitat on shaping wing morphology in a large dataset of over 3500 individuals, representing 13 *Heliconius* species from across the Neotropics. We find that both larval and adult behavioral ecology correlate with patterns of wing sexual dimorphism and adult size. Species with solitary larvae have larger adult males, in contrast to gregarious *Heliconius* species, and indeed most Lepidoptera, where females are larger. Species in the pupal‐mating clade are smaller than those in the adult‐mating clade. Interestingly, we find that high‐altitude species tend to have rounder wings and, in one of the two major *Heliconius* clades, are also bigger than their lowland relatives. Furthermore, within two widespread species, we find that high‐altitude populations also have rounder wings. Thus, we reveal novel adaptive wing morphological divergence among *Heliconius* species beyond that imposed by natural selection on aposematic wing coloration.

Identifying the selective forces driving phenotypic divergence among closely related species lies at the core of evolutionary biology research. Adaptive radiations, in which descendants from a common ancestor rapidly fill a variety of niches, are ideal systems to investigate morphological divergence (Schluter 2000). The study of adaptive radiations has revealed that evolution often comes up with similar solutions for similar problems at the phenotypic and genetic levels (Losos 2010; Marques et al. 2019). Speciose groups that have repeatedly and independently evolved convergent adaptations to life‐history strategies and environments are good systems in which study selection drivers (Schluter 2000). Nevertheless, adaptive phenotypic evolution is often complex and multifaceted, with more than a single selective force in action (Maia et al. 2016; Nosil et al. 2018). For example in birds, sex differences in plumage coloration are driven by intraspecific sexual selection, while natural selection drives sexes toward more similar colorations (Dunn et al. 2015). Integrative approaches that make use of tractable traits across well‐resolved phylogenies are needed to explore the selective forces driving phenotypic evolution.

Butterfly wing coloration has been the focus of considerable research effort and major strides have been made toward understanding how and when evolution leads to complex wing color patterns, conferring aposematism, camouflage, or a mating advantage (Merrill et al. 2012; Chazot et al. 2016; Nadeau et al. 2016). The dazzling diversity of butterfly color patterns among species has perhaps obscured the less conspicuous phenotypic diversity of wing shapes and sizes, which are more often regarded as the result of sexual selection, flight trade‐offs or developmental constraints (Singer 1982; Allen et al. 2011), rather than drivers of local adaptation and species diversification (Srygley 2004a; Cespedes et al. 2015; Chazot et al. 2016). A recent review assessing the ecology of butterfly flight, identified habitat, predators, and sex‐specific behaviors as the selection forces most likely driving wing morphology variation, but highlighted the need for further phylogenetic comparative studies that identify the adaptive mechanisms shaping wings (Le Roy et al. 2019).

Differences in behavior between sexes have been identified as one of the main drivers of wing aspect ratio and size sexual dimorphism in insects (Rossato et al. 2018a; Le Roy et al. 2019). In butterflies, males tend to spend more time looking for mates and patrolling territories, while females focus their energy on searching for suitable host plants for oviposition (Rossato et al. 2018b). The same wing trait can be associated with different life history traits in each sex, resulting in sex‐specific selection pressures. For example, in the Nearctic butterfly *Melitaea cinxia*, wing aspect ratio only correlates with dispersal in females, as males experience additional selection pressures that counteract selection for dispersal wing phenotypes (Breuker et al. 2007). Sex‐specific behaviors can impact wing aspect ratio and size, but differences in life histories, even across closely related species, could also have large impacts on the strength and direction of these effects (Cespedes et al. 2015; Chazot et al. 2016).

Another important source of phenotypic variation in insect wings is the physical environment they inhabit throughout their range. Air pressure decreases with altitude, which in turn reduces lift forces required for flight. To compensate for this, insects may increase wing area relative to body size to reduce the velocity necessary to sustain flight (Dudley 2002; Dillon et al. 2018). Wing aspect ratio in *Drosophila melanogaster* has been observed to vary adaptively across latitudes and altitudes, with wings getting rounder and larger in montane habitats, possibly to maintain flight function in lower air pressures (Stalker and Carson 1948; Pitchers et al. 2012; Klepsatel et al. 2014).

In butterflies, high aspect ratios, that is, long and narrow wings, reduce drag caused by wing tip vortices, thus lowering the energy required for flight and promoting gliding for longer distances (Le Roy et al. 2019). Variation in wing phenotypes can occur at the microhabitat level, for example, *Morpho* butterfly clades in the understory have rounder wings than canopy‐specialist clades, presumably for increased maneuverability (Chazot et al. 2016). An extreme case of environmental effects on wing morphology can be found in Lepidoptera inhabiting the windy, barren highlands of the Andes, where an interaction between behavioral sex differences and extreme climatic conditions have led to flightlessness in females of several species (Pyrcz et al. 2004).

*Heliconius* is a genus of Neotropical butterflies that has been studied for over two centuries with a well resolved phylogeny (Kozak et al. 2015, 2018). It represents a striking case of Müllerian mimicry, with co‐occurring subspecies sharing warning wing color patterns to avoid predators and leading to multi‐species mimicry rings across South America (Merrill et al. 2015). Wing aspect ratio and size are part of the mimetic signal (Jones et al. 2013; Mérot et al. 2016; Rossato et al. 2018a). Wing morphology is involved in many aspects of *Heliconius* biology other than mimicry, such as mating or flight mode, but these have been less well studied (Rodrigues and Moreira 2004; Srygley 2004a; Mendoza‐Cuenca and MacÍas‐Ordóñez 2010). As the only butterflies that pollen‐feed, their long life‐spans and enlarged brains allow them to memorize foraging transects that are repeated daily following a short dispersal post‐emergence phase of up to 1.5 km (Cook et al. 1976; Jiggins 2016).

Larval gregariousness has evolved independently three times across the phylogeny, with some species laying clutches of up to 200 eggs, while others lay eggs singly and larvae are often cannibalistic (Beltrán et al. 2007). Gregarious *Heliconius* species would be predicted to have larger sized females to carry the enlarged egg load, as is the case with most Lepidoptera (Allen et al. 2011). Another striking life history trait is pupal‐mating, which is only found in one of the two major clades (hereafter the “erato clade”), having arisen following the most basal split in the *Heliconius* phylogeny. This mating strategy involves males copulating with females as they emerge from the pupal case (Deinert et al. 1994; Beltrán et al. 2007). Pupal‐mating leads to a whole suite of distinct selection pressures but these are hard to tease apart from the effects of phylogeny due to its single origin (Beltrán et al. 2007; Thurman et al. 2018). Further ecological differences could arise from adaptation to altitude. Some species are relatively high‐altitude specialists, such as *H. telesiphe* and *H. hierax* found above 1000 m, while others range widely, such as *H. melpomene* and *H. erato*, which can be found from 0 to 1800 m above sea level (Rosser et al. 2015; Jiggins 2016). Potential adaptations to altitude are yet to be explored.

The wide range of environments that *Heliconius* species inhabit, together with their diverse natural history and well‐resolved phylogeny, make them a good study system for teasing apart the selective forces driving wing phenotype (Merrill et al. 2015; Jiggins 2016). Here, we examine variation in wing aspect ratio and size across 13 species that span most of the geographical range of the *Heliconius* genus. First, we photographed thousands of wings collected by many *Heliconius* researchers since the 1990s from wild populations across South and Central America, covering a 2100 m elevation range (Fig. 1A). Wing dimensions for 3515 individuals, obtained with an automated pipeline and standardized images, were then used to address the following questions. (1) Are there size and aspect ratio sexual dimorphisms, and if so, do they correlate with known life‐history traits? (2) To what extent are wing aspect ratio and size variation explained by shared ancestry? (3) Are wing aspect ratio and size affected by the elevations species inhabit?

**Figure 1:**
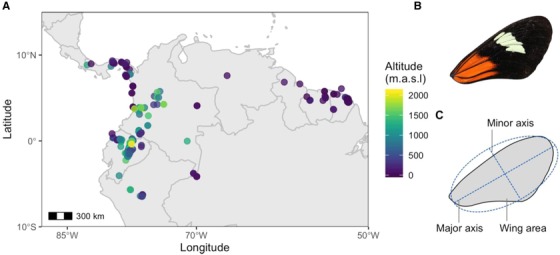
Localities and forewing measurements. (A) Map of exact locations (*n* = 313) across South America from where the samples used for our analyses were collected. Points are colored by altitude. (B) Representative of a right forewing image of *H. melpomene malleti*. (C) Measurements taken from each wing by fitting an ellipse with Fiji custom scripts. [Color figure can be viewed at http://wileyonlinelibrary.com]

## Methods

### STUDY COLLECTION

The wild specimens studied here were collected using hand nets between 1998 and 2018 in 313 localities across Panama, Colombia, Ecuador, French Guiana, Suriname, and Peru (Fig. 1A), and stored in the Department of Zoology, University of Cambridge (Earthcape database). Collection altitudes ranged from sea level to 2100 m above sea level (Fig. 1A). Detached wings were photographed dorsally and ventrally with a DSLR camera with a 100 mm macro lens in standardized conditions. All the images are available in the public repository Zenodo (https://zenodo.org/communities/butterfly/) and full records with data are stored in the EarthCape database (https://heliconius.ecdb.io).

### WING MEASUREMENTS

Damage to wings was manually scored in all the images and damaged specimens were excluded from our analyses. To obtain wing measurements from the images, we developed custom scripts for Fiji (Schindelin et al., 2012), to automatically crop, extract the right or left forewing, and perform particle size analysis (Fig. 1B). Butterflies predominantly use their forewings for flight (Wootton 1992; Le Roy et al. 2019) and hindwings tend to be more damaged in Heliconius due to in‐flight predation and fragile structure, thus we only include forewings here. Forewing and hindwing areas are tightly correlated in this genus (Strauss, 1990). For wing area, we obtained total wing area (in mm^2^, hereafter “size”).

For examining wing aspect ratio, the custom scripts first fitted an ellipse to the forewings and measured the length of the longest axis and the length of the axis at 90° to the former (Fig. 1C). Aspect ratio corresponds to the length of the major axis divided by the length of the minor axis, hereafter “aspect ratio” (Fig. 1C). Aspect ratio was used as a proxy of wing shape, as it has been widely used in previous studies and correlates to gliding efficiency (Le Roy et al. 2019). The data were checked for visual outliers on scatter‐plots, which were examined, and removed from the analyses if the wing extraction pipeline had failed.

### STATISTICAL ANALYSES

All analyses were run in R version 2.13 (R Development Core Team 2011) and graphics were generated with the package *ggplot2* (Ginestet 2011). Packages are specified below. All R scripts can be found in the public repository Zenodo (Zenodo: https://doi.org/10.5281/zenodo.3491029), including custom Fiji scripts for wing image analysis. Species and sexes mean trait values were calculated for the 13 *Heliconius* species in our study. Each species had more than 30 individuals and all individuals had accurate locality and altitude data (Table S1), resulting in a dataset of 3515 individuals.

#### Sexual dimorphism across species

Sexual dimorphism in wing area and aspect ratio was estimated as the female increase in mean wing area and aspect ratio with respect to males, thus negative values represent larger trait values in males, while positive values represent larger trait values in females. Pairwise *t*‐tests were used to estimate the significance of sexual size/shape dimorphism in each species.

We modeled variation in wing area and aspect ratio sexual dimorphism across species with ordinary least squares (OLS) linear regressions, implemented in the ‘lm’ function. For models of sexual wing area and aspect ratio sexual dimorphism, predictor variables initially included larval gregariousness of the species (gregarious or solitary, as classified in Beltrán et al. 2007), mating strategy (pupal‐mating vs. adult‐mating clade), species mean wing aspect ratio and area, and species wing aspect ratio or size sexual dimorphism (respectively). Wing size sexual dimorphism had a marginally significant phylogenetic signal (Abouheif Cmean = 0.25, *P* = 0.05), so we present the sexual size dimorphism model incorporating phylogeny as correlation term in the Supporting Information (Tables S3 and S4). We used backward selection with Akaike Information Criterion corrected for small sample sizes (AICc, Hurvich and Tsai 1989) where the best models had the lowest AICc values, implemented with the package MuMin (Bartón 2019). We report the overall variation explained by the fitted linear models (*R* ^2^) and the relative contributions of each explanatory variable (partial *R* ^2^), estimated with the package *relaimpo* (Grömping 2006).

#### Variation across species

To test whether variation in wing aspect ratio and area across species was constrained by shared ancestry, we calculated the phylogenetic signal index Abouheif's Cmean (Abouheif 1999), which is an autocorrelation metric suitable for datasets with a relatively low number of species and that does not infer an underlying evolutionary model (Münkemüller et al. 2012). Observed and expected distribution plots for phylogenetic signal estimates are shown in the Supporting Information and were computed with the package *adephylo* (Jombart and Dray 2010). We used a pruned tree with the 13 species under study from the most recent molecular *Heliconius* phylogeny (Kozak et al., 2015). We plotted centered trait means across the phylogeny with the function barplot.phylo4d() from the package *phylosignal* (Keck et al. 2016). To test and visualize phylogenetic signal further, we built phylocorrelograms for each trait with the function phyloCorrelogram() of the same package, which estimates Moran's I autocorrelation across matrices with varying phylogenetic weights. Then, the degree of correlation (Morans’ I) in species trait values can be assessed as phylogenetic distance increases (Keck et al. 2016).

To study variation in wing area and aspect ratio across species, we took a phylogenetic comparative approach. These methods assume that species‐specific mean trait values are a good representation of the true trait values of the species under study; in other words, the within‐species variation is negligible compared to the across‐species variation (Garamszegi 2014). To test this, we first used an ANOVA approach, with species as a factor explaining the variation of mean trait values. We then estimated within‐species trait repeatability, or intraclass correlation coefficient (ICC), with a linear mixed model approach. This requires the grouping factor to be specified as a random effect, in this case species, with a Gaussian distribution and 1000 parametric bootstraps to quantify uncertainty, implemented with the function rptGaussian() in *rptR* package (Stoffel et al. 2017). By specifying species as a random effect, the latter approach estimates the proportion of total trait variance accounted for by differences between species. A trait with high repeatability indicates that species‐specific trait means are reliable estimates for further analyses (Stoffel et al. 2017). We, nevertheless, accounted for within‐species variation in the models described below.

To test the effect of altitude on wing aspect ratio and size across species, we used a phylogenetic generalized least squares (PGLS) approach. Species wing trait means may be correlated due to shared ancestry (Freckleton et al. 2002; Chazot et al. 2016). Therefore, to explore the effects of the environment on the traits under study, models that incorporate expected correlation between species are required, such as PGLS. Although often ignored, these models assume the presence of phylogenetic signal on the model residuals of the trait under study (here wing aspect ratio or size) controlling for covariates that affect the trait mean (allometry, sex ratio), and not just phylogenetic signal on the species mean trait values (Revell 2010; Garamszegi 2014). Thus, to check if this assumption was met, we estimated phylogenetic signal as described above (Keck et al. 2016) for the residuals of a generalized least squares (GLS) of models that had wing aspect ratio or size as response variables, and the size and aspect ratio (respectively) and sex ratio as explanatory variables, to ensure this assumption of PGLS model was met. To visually inspect phylogenetic sinal on the residuals, we obtained phylogenetic correlograms for these and centered trait residuals for plotting across the phylogeny as detailed above for trait means (presented in the Figs. S3 and S4; Keck et al. 2016).

Significant phylogenetic signal was detected in mean wing size and in the residuals of both traits, wing aspect ratio, and area regression models ( Figs. S4 and S5), so we used maximum log‐likelihood PGLS regression models with the phylogenetic correlation fitted as a correlation term, implemented with the gls() function from the *nmle* package (Pinheiro et al. 2007). We assumed a Brownian motion model of trait evolution for both traits, by which variation across species accumulated along all the branches at a rate proportional to the length of the branches (Freckleton et al. 2002). To select the most supported model given the available data, that is, one that improves model fit while penalizing complexity, we used the Aikaike Information Criteria corrected for small sample sizes (AICc, Hurvich and Tsai 1989), where the best models had the lowest AICc values, implemented with the package MuMin (Bartón 2019). Maximal PGLS models included species mean altitude and distance from the Equator (to control for potential latitudinal clines), sex ratio in our samples interacting with either wing aspect ratio or wing size, to control for potential allometric and sexual dimorphism relationships, which could be different among closely related taxa (Outomuro and Johansson 2017). Most species are found in the Andean mountains or the Amazonian region near the Equator, so we did not have much power to examine variation with latitude in wing aspect ratio and size across species, but we included distance from the Equator as an explanatory variable in the PGLS models to account for it. Minimal PGLS models consisted of the trait under study explained solely by its intercept, without any fixed effects. All model selection tables can be found in the Supporting Information (Tables S3 and S5). Finally, we weighted PGLS regressions to account for unequal trait variances and unbalanced sample sizes across species (for sample sizes and standard errors of species’ trait means see Table S1). This was achieved by modifying the error structure of the model with combined variances obtained with the function varFixed() and specified with the argument “weights” (Pinheiro et al. 2007; Paradis 2012; Garamszegi 2014). In this study, 74.8% of the individuals were collected in the last 10 years, thus we did not have power to detect any changes in wing morphology across species potentially incurred by climate change (Fig. S1). Future studies could focus on temporal changes in wing morphology in areas and species that have been well sampled throughout the years.

#### Variation within species

We selected the two most abundant and geographically widespread species within our dataset, *H. erato* (*n* = 1685) and *H. melpomene* (*n* = 912; Table S1), to examine variation in wing area and aspect ratio within species. We modeled variation in size and aspect ratio with ordinary least squares (OLS) linear regressions for each species, implemented in the ‘lm’ function. For all models, predictor variables initially included the terms altitude, distance from the Equator, longitude, aspect ratio or wing area, and sex, as well as the plausible interactions between them (Table S5). We then used step backward and forward selection based on AIC with the function stepAIC(), from the *MASS* package (Ripley, 2011; Zhang, 2016; full models and model selection tables in Tables S5 and S6).

## Results

We obtained intact‐wing measurements for 3515 individuals of 13 *Heliconius* species from across the phylogeny and from over 350 localities (Fig. 1; Table S1). We have made all of these wing images publicly available at the Zenodo repository.

### SEXUAL DIMORPHISM

**Figure 2:**
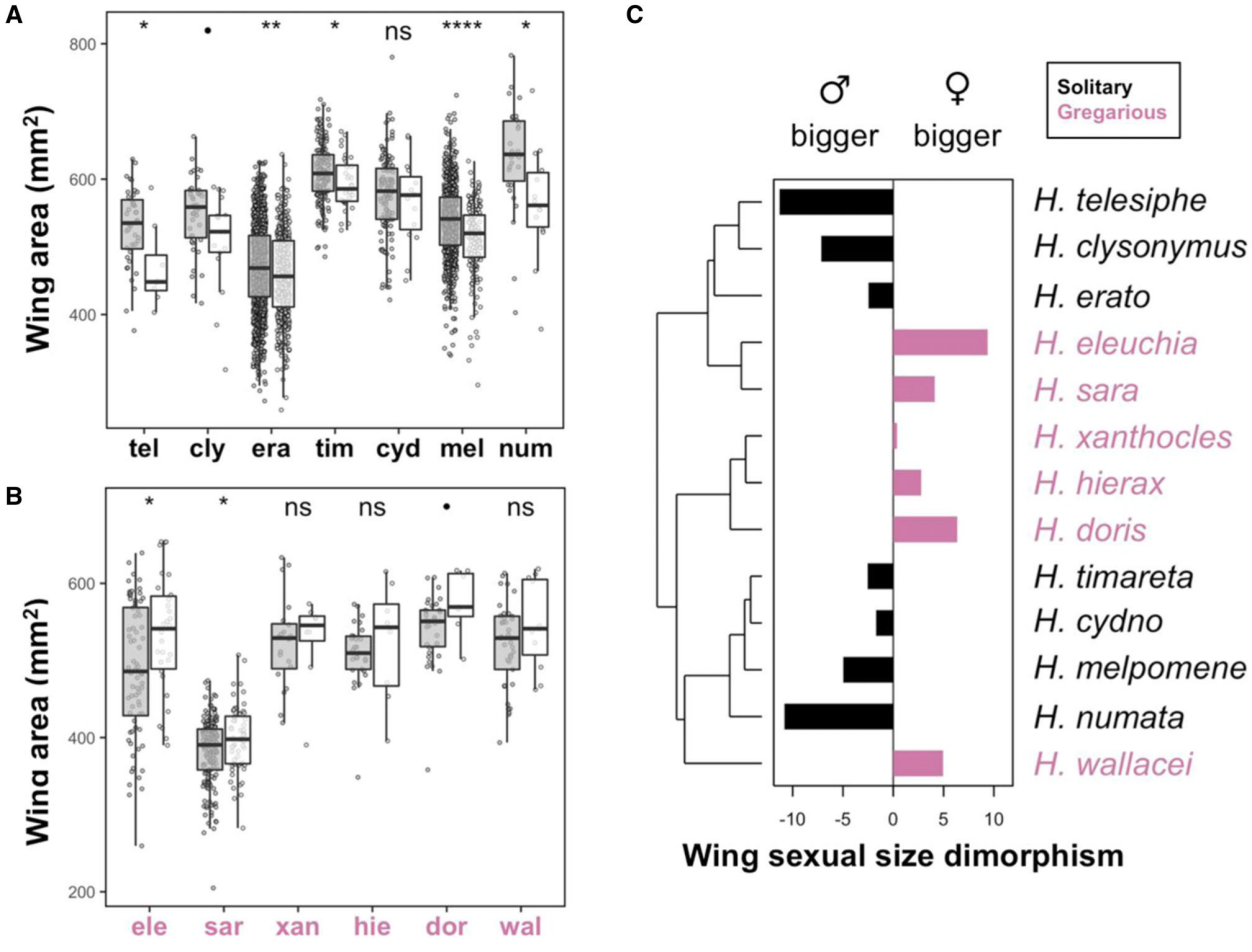
Sexual wing area dimorphism across species and the phylogeny. (A) Wing size differences between males (grey) and females (white) of the seven single egg‐laying species and (B) the six gregarious species in this study. Error bars represent 95% confidence intervals of the means. Stars represent significance levels of two sample *t*‐tests between female and male wing areas for each species (•<0.1, ^*^< 0.05, ^**^<0.01, ^***^<0.001); for full *t*‐tests output, see Table S1. (C) Bar plot represents sexual size dimorphism calculated as percentage difference in female versus male size (positive means bigger females, right panel). Species with gregarious larvae are colored in pink, and those with solitary larvae are colored in black. [Color figure can be viewed at http://wileyonlinelibrary.com]

Sexual dimorphism in wing area was found throughout the phylogeny, but in opposing directions in different species (Fig. 2). Mean sizes were significantly or marginally significantly different among sexes in nine species, all of which were represented by more than 40 individuals (Table S2 for two sample *T*‐test summary statistics), indicating that the nonsignificant trends in other species probably reflect a lack of power caused by low numbers of females typically collected in the wild (Table S1). The six species with trends toward larger females have gregarious larvae (pink, Fig. 2), whereas the seven species with trends toward larger males lay eggs individually (black, Fig. 2). Larval gregariousness alone explained 69% of the total natural variation in sexual size dimorphism across species (Table 1; Gaussian LM: F_1,11_ = 27.2, *P* < 0.001, *R* ^2^ = 0.69). There was a marginally significant phylogenetic signal in sexual size dimorphism (Abouheif's Cmean = 0.24, *P* = 0.05; Fig. S3), so we repeated the analysis accounting for phylogeny and the results are presented in the Supporting Information. This would be expected from the evolutionary history of gregariousness, as it is present in all species of three lineages that are well represented in our study (Beltrán et al. 2007). However, when accounting for phylogenetic correlation in the model larval gregariousness remained a significant predictor of size sexual dimorphism (Table S4).

**Table 1:** Summary of model outputs derived from the interspecific analyses of sexual size dimorphism, wing aspect ratio and area phylogenetic generalized least squares. For the latter, summary statistics are presented for the overall model and the explanatory variable of interest, altitude. Full model tables for PGLS can be found in the Supporting Information Materials (Table S2)

Response variable (wing trait)	Trait repeatability (R)	Model type	Corr. structure	Fixed effects	Estimation	SE	*t*‐Value	*P*‐value	*df* (res)	Adjusted *R* ^2^
Sexual size dim.	NA	lm	Gaussian	(Intercept)	4.72	1.35	3.5	0.004***	13 (11)	–
				Solitary larvae	−10.5	2.0	−5.2	0.0001***		0.69
Wing shape	0.74	PGLS	Phylogenetic,	(Intercept)	0.15	0.53	0.27	0.79	13 (9)	NA
(aspect ratio)	(*P* = 0)	(nmle)	intrasp. variance, sample size	Altitude	−1.5 × 10^–4^	6.3 × 10^–5^	−2.4	0.040*		
Wing size (area)	0.48 (*P* = 0)	PGLS	Phylogenetic,	(Intercept)	474.52	75.37	6.30	0.00	13 (8)	NA
		(nmle)	intrasp. variance, sample size	Altitude	0.16	0.04	3.47	0.008**		

Sexual dimorphism in wing aspect ratio was found in three species (Fig. S4), *H. erato* and *H. wallacei* had longer winged males whereas the high‐altitude specialist *H. eleuchia* had longer winged females (Table S2, *T*‐test, *H. erato*: *t*_843_ = 10.4, *P* < 0.0001, *H. eleuchia*: *t*_49_ = –2.3, *P *< 0.05, *H. wallacei*: *t*_19_ = 2.2, *P* < 0.05). Wing aspect ratio sexual dimorphism across species could not be explained with the variables here studied and had no phylogenetic signal (Abouheif's Cmean = –0.02, *P* = 0.3; Fig. S3).

### PHYLOGENETIC SIGNAL

The 13 *Heliconius* species studied differed significantly in wing area and aspect ratio (ANOVA, aspect ratio: F_12, 3502_ = 228.4, *P* < 0.0001, area: F_12, 3502_ = 216.4, *P* < 0.0001; Tukey‐adjusted comparisons Fig. S2). We estimated within‐species trait repeatability to assess their reliability as species mean estimates for phylogenetic analyses. Wing aspect ratio had higher intra‐class repeatability than wing area, with 74% and 48% of the total aspect ratio and size variance explained by differences between species, respectively (aspect ratio: *R* = 0.74, SE = 0.09, *P* < 0.0001; size: *R* = 0.48, SE = 0.1, *P* < 0.0001). We estimated intraclass repeatability for males and females separately to remove the potential effect of size sexual dimorphism on trait variation, and male size repeatability remained much lower than male wing aspect ratio repeatability (male aspect ratio: *R* = 0.75, SE = 0.08, *P* < 0.0001; male size: *R* = 0.53, SE = 0.1, *P* < 0.0001). Females had the same wing aspect ratio repeatability as males, whereas wing size repeatability was lower for females probably due to smaller sample sizes (Female aspect ratio: *R* = 0.75, SE = 0.05, *P* < 0.0001; female size: *R* = 0.44, SE = 0.1, *P* < 0.0001).

**Figure 3:**
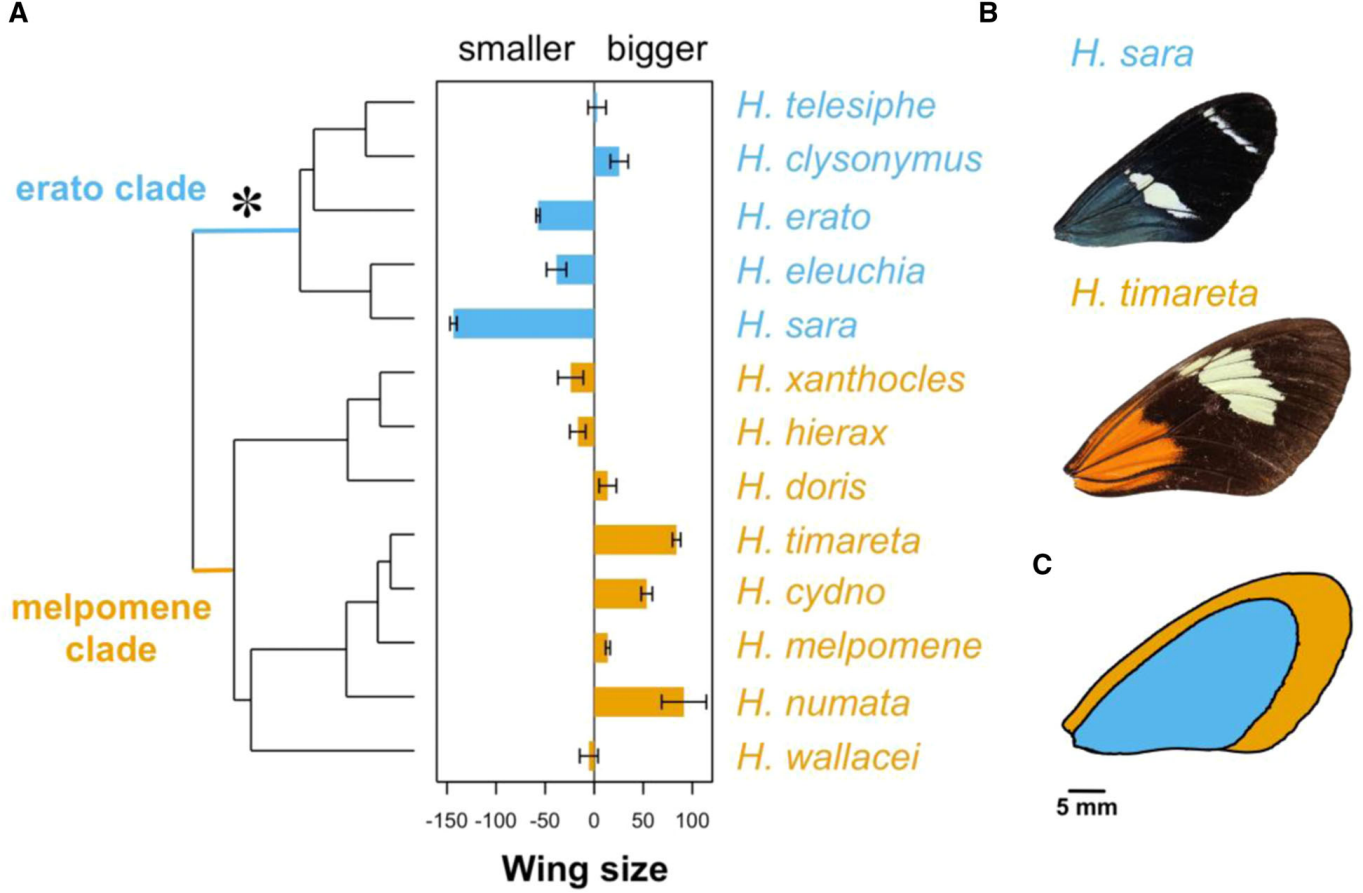
Male wing area differences across the phylogeny. (A) Bar plot represents centered mean wing area per species (positive values represent species with bigger wings than the average *Heliconius* wing). Wing area, *x*‐axis, is the difference in wing area from the mean (in mm^2^). Error bars represent standard errors. The star represents the origin of pupal‐mating. Species from the erato clade are in blue, and those from the melpomene clade are in orange. (B) Representatives of *H. timareta* and *H. sara* closest to the mean wing area of the species are shown (606.25 and 386.6 mm^2^, respectively). (C) Images from (B) superimposed to compare visually the mean size difference between the two species. [Color figure can be viewed at http://wileyonlinelibrary.com]

Mean wing aspect ratio showed no phylogenetic signal (Abouheif's Cmean = 0.15, *P* = 0.1; Figs. S3 and S5B); in other words, closely related species were not more similar to each other than to distant ones. In contrast, mean wing area showed a strong phylogenetic signal, by which phylogenetically closely related species were more likely to have similar wing areas (Fig. 3, Abouheif's Cmean = 0.33, *P* = 0.01; Figs. S3 and S6A and B). Wing areas of species in the melpomene clade were on average 14.8% larger than those of species in the erato clade, with *H. timareta* being 64% larger than *H. sara* (Fig. 3, *H. timareta*: mean = 606.6 mm^2^, SE = 3.1; *H. sara*: mean = 387 mm^2^, SE = 2.9). Nevertheless, when controlling for sex ratios and allometry on the traits under study, wing aspect ratio and size, the residuals of both traits show a strong phylogenetic signal (Figs. S5 and S6A and C; aspect ratio residuals: Abouheif's Cmean = 0.42, *P* < 0.001; Fig. S3A and C; size residuals: Abouheif's Cmean = 0.44, *P* < 0.001). These results support the use of phylogenetic models to study variation in wing aspect ratio and size across species.

### PATTERNS ACROSS SPECIES AND ALTITUDES

**Figure 4:**
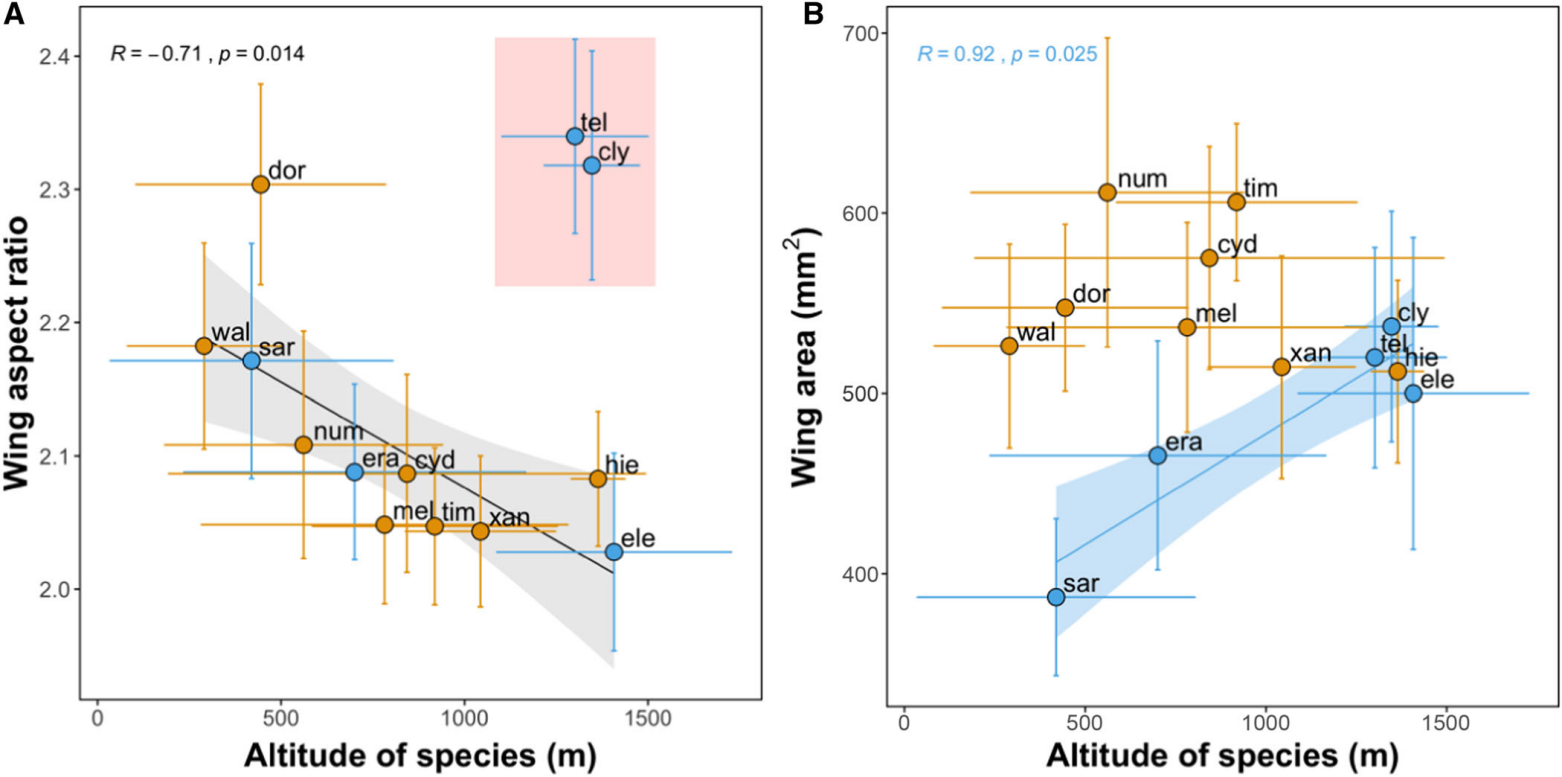
Species variation in wing aspect ratio (A) and wing area (B). Plots show the effect of altitude (meters above sea level) on wing aspect ratio (major axis/minor axis, higher values represent longer wings) and wing area (mm^2^). Points represent species mean raw values per species. Horizontal and vertical lines show standard error for species mean altitude and mean trait, respectively. Lines show best linear fit and are colored by clade when clade was a significant predictor (blue: erato clade, orange: melpomene clade). Shaded areas show confidence bands at 1 standard error. The point labels correspond to the first three characters of the following *Heliconius* species: *H. telesiphe*, *H. clysonymus*, *H. erato*, *H. eleuchia*, *H. sara*, *H. doris*, *H. xanthocles*, *H. hierax*, am*H. wallacei, H. numata*, *H. melpomene*, *H. timareta*, and *H. cydno*. Two species, *H. telesiphe* and *H. clysonymus*, showed high levels of phylogenetic autocorrelation (Fig. S7) and were thus excluded from the linear model plotted (but not from the main analyses where phylogeny is accounted for). [Color figure can be viewed at http://wileyonlinelibrary.com]

Species mean altitude had an effect on wing area and aspect ratio (Table 1). Species wings got rounder, that is, lower aspect ratios, with increasing altitudes both when accounting for fixed effects and the phylogeny (Table 1; for full model, see Table S4). These patterns were also evident when examining raw mean wing aspect ratios (Fig. 4A, Gaussian LM: F_1, 9_ = 5.37, *P* < 0.05, *R* ^2^ = 0.30), except in the *H. telesiphe* and *H. clysonymus* highlands clade, which showed significant phylogenetic autocorrelation (Moran's *I* index: *H. clysonymus* 0.53, *H. telesiphe* 0.49). Species wings got larger with elevation (Table 1; for full model, see Table S4). Without accounting for phylogeny or any fixed effect this is only evident in the erato clade, where high altitude species were bigger than their lowland sister species (Fig. 4B, blue, Gaussian LM: F_1,10_ = 17.1, *R* ^2^ = 0.80, *P* = 0.03). However, when assessing individuals from all species together, it becomes clear that larger individuals of both clades tend to be found at higher altitudes (Fig. S8). Both wing size and wing aspect ratio were also significantly correlated with distance from the Equator, and wing aspect ratio was affected by species sex ratio too (Table S4).

### PATTERNS WITHIN SPECIES AND ACROSS ALTITUDES

**Figure 5:**
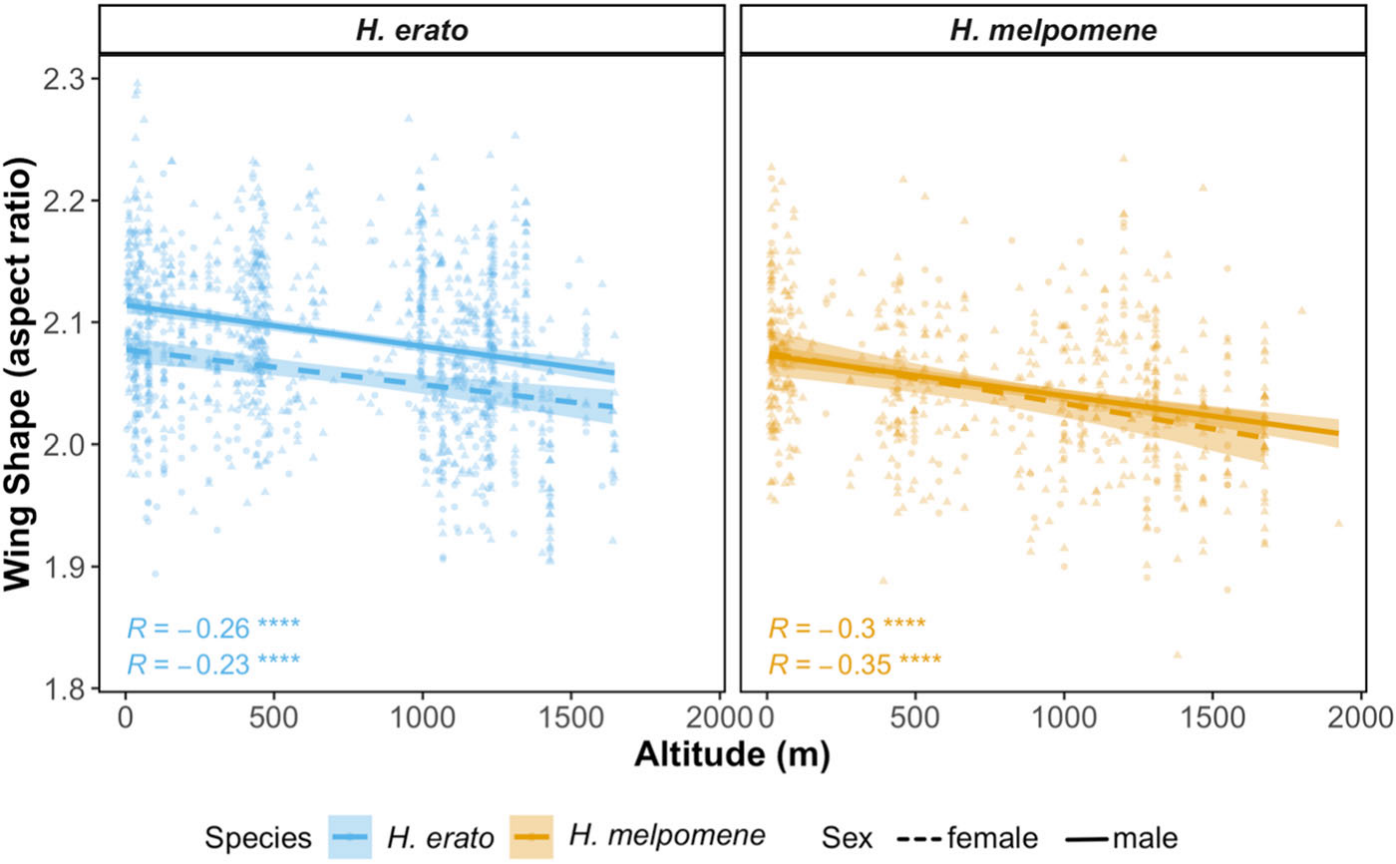
Within‐species variation in wing aspect ratio across altitudes in *H. erato* (blue) and *H. melpomene* (orange), females (triangles, dotted line) and males (circles, solid line). Lines show best linear fit and are colored by species. Shaded areas show confidence bands at 1 standard error. Pearson correlation coefficients and *P*‐values are shown for each regression plotted. (•<0.1, ^*^ < 0.05, ^**^ <0.01, ^***^ <0.001). [Color figure can be viewed at http://wileyonlinelibrary.com]

Wings got rounder (lower aspect ratio) with increasing altitude in *H. erato* and *H. melpomene* (Fig. 5, *H. erato*: Gaussian LM: F_6, 1296_ = 32.7, *P* < 0.001, *R* ^2^ = 0.13; *H. melpomene*: Gaussian LM: F_6, 673_ = 20.1, *P* < 0.001, *R* ^2^ = 0.14). Individual altitude was the strongest predictor of wing aspect ratio for both species, with sex and wing area being second best in *H. erato* and *H. melpomene*, respectively (Table S6 and Fig. S13A and B; Fig. 5). Conversely, the relative importance of explanatory variables of wing area varied for each species (Table S6 and Fig. S13A and B; Fig. 5), and the *H. erato* model explained less of the overall variation in wing area (Fig. S11, *H. erato*: Gaussian LM: F_7,1295_ = 9.36, *P* < 0.001, *R* ^2^ = 0.04, *H. melpomene*: Gaussian LM: F_7, 672_ = 23.06, *P* < 0.001, *R* ^2^ = 0.18). Wing area in *H. erato* was correlated with allometric factors interacting with altitude, whereas wing area in *H. melpomene* was correlated with distance from the Equator (Table S6 and Figs. S10 and S13C and D). Wing area and aspect ratio differed among co‐mimicking races of *H. erato* and *H. melpomene*, despite inhabiting the same geographic areas (Fig. S12).

## Discussion

The fascination for butterfly wing coloration has stimulated many generations of research and *Heliconius* wing patterns have proven to be excellent study systems for understanding evolution and speciation. Here, we have extended this research by examining wing shape and size variation among more than 3500 individual butterflies, across sexes, clades, and altitudes in 13 species of *Heliconius* butterflies. We have shown that a large proportion of female biased sexual size dimorphism can be explained by the evolution of larval gregariousness, and that male biased sexual size dimorphism is present only in species that lay eggs individually, regardless of their mating strategy. For the first time in this system, we describe wing morphological variation across environmental clines, with species and populations found at higher altitudes consistently having rounder wings. Here, we demonstrate that *Heliconius* wing area and aspect ratio are potentially shaped by a plethora of behavioral and environmental selection pressures, in addition to those imposed by Müllerian mimicry.

### WING ASPECT RATIO VARIATION

Wing aspect ratio in butterflies and other flying animals determines flight mode and speed (Farney and Fleharty 1969; Buler et al. 2017), and is therefore predicted to vary with life‐history requirements across sexes and species. Despite being a simple descriptor of wing shape, aspect ratio has been demonstrated to correlate functionally with gliding efficiency in butterflies by increasing lift‐to‐drag ratios (Ortega Ancel et al. 2017; Le Roy et al. 2019). Long wings are generally associated with faster gliding flying, whereas round wings with low aspect ratio values favor slow but more maneuverable flight motions (Betts and Wootton 1988; Chai and Srygley 1990; Chazot et al. 2016; Le Roy et al. 2019). For instance, monarch butterfly populations with longer migrations have more elongated wings than resident populations (Satterfield and Davis 2014), and males of *Morpho* species that dwell in the canopy also have higher aspect ratios to glide faster through open areas (DeVries et al. 2010). In contrast, female *Morpho* butterflies tend to have rounder wings, and aspect ratio sex differences are stronger in species with colour dimorphism, as varying crypsis may require specific flight behaviors (Chazot et al. 2016).

*Heliconius* are not notoriously sexually dimorphic especially when compared to other butterflies such as *Morpho* (Chazot et al. 2016; Jiggins 2016). However, there are important behavioral differences between the sexes. Females are thought to have different flight habits, as they spend much of their time looking for specific host plants for oviposition (Dell'Aglio et al. 2016), or precisely laying eggs on suitable plants, while males tend to patrol open areas searching for receptive females and visit flowers more often (Joron 2005; Jiggins 2016). Thus, it might be predicted that females should have lower aspect ratios, that is, rounder wings, than males (Jones et al. 2013). However, we only found three species with significant, but opposing, sexually dimorphic wing aspect ratios. The wings of males in *H. erato* were longer than the wings in females, whereas male *H. eleuchia* and *H. wallacei* had rounder wings than those of females (Fig. S3). *Heliconius* wing shape sex differences may require multivariate descriptors of wing morphology and/or analysis of the hindwings, which possess the pheromone‐dispersing androconial patch in males (Jones et al. 2013; Mérot et al. 2013, 2016). In addition, the relatively low collection numbers of female *Heliconius* could hinder the detection of subtle wing aspect ratio differences across the sexes.

Sexual selection has long been known to affect wing color pattern in *Heliconius*, as it is used for mate recognition and choice (Merrill et al. 2012). More recently, wing aspect ratio has been shown to be part of the mimetic warning signal in *Heliconius* and their co‐mimics (Jones et al. 2013), as it determines flight motion and defines the overall appearance of the butterfly (Srygley 1994, 2004a). For instance, wing aspect ratios between two different morphs of *H. numata* differed consistently across their overlapping ranges, in parallel with their respective and distantly related *Melinea* co‐mimics (Jones et al. 2013). Within‐morph wing aspect ratio variation was observed across the altitudinal range of *H. timareta* in Peru (Mérot et al. 2016), and in the *Heliconius* postman mimicry ring in Brazil significant across‐species wing aspect ratio differences were also found (Rossato et al. 2018a). These studies highlight that while it is clear that color pattern and, to some extent, flight are important for mimicry in *Heliconius*, wing aspect ratio is also subject to other selection pressures (Mérot et al. 2016; Rossato et al. 2018b).

We found that species inhabiting higher altitudes tend to have rounder wings, after accounting for phylogeny, sample size, and intraspecific variance (Fig. 4A), except in the *H. telesiphe*–*H. clysonymus* clade. The latter species may require morphometric analyses of wing tip shape alone, as the overall wing morphology differs significantly from the rest of the *Heliconius* species here studied (Fig. S7). Interestingly, these patterns were maintained within‐species, with high‐altitude populations of *H. erato* and *H*. melpomene having lower aspect ratios (Fig. 5). Furthermore, altitude was the best predictor of wing aspect ratio in both species (Fig. S13). Rounder wings aid maneuverability and are associated with slower flight in butterflies (Berwaerts et al. 2002; Le Roy et al. 2019) and slower flights are generally associated with a decrease in ambient temperature (Gilchrist et al. 2000). In addition, air pressure, which directly reduces lift forces required to offset body weight during flight (Dillon 2006), decreases approximately 12% across the mean altitudinal range of the species here studied. Thus, the rounder wings in high altitude *Heliconius* species and populations may aid flying in dense cloud forests, where increased maneuverability could be beneficial, or compensating for lower air pressure at higher altitude.

### WING AREA VARIATION

Wing area showed significant sexual dimorphism in more than half of the species studied here, but some species had larger males and others larger females (Fig. 2). In most butterflies, females are overall larger than males, presumably because fecundity gains of increased body size are greater for females (Allen et al. 2011). Larger wings are required to carry larger and heavier bodies, and so Lepidoptera females also tend to have larger wings (Allen et al. 2011; Le Roy et al. 2019). Indeed, in this study, the *Heliconius* species with larger‐winged females were those that lay eggs in large clutches and that have highly gregarious larvae (Fig. 2, Beltrán et al. 2007). A recent study on two species not included here reported wing size dimorphism with larger females in the gregarious *H. eratosignis ucayalensis* and larger males in the single‐egg layer *H. demeter joroni* (Rosser et al. 2019). Thus, females of these species are likely investing more resources in fecundity than males, which leads to larger body and wing sizes that allow them to carry and lay eggs in clutches throughout adulthood. Larval development time correlates with adult size in *H. erato* (Rodrigues and Moreira 2002) and growth rates seem to be the same across sexes, at least in the gregarious *H. charithonia* (Kemp 2019), so we hypothesize that females take longer to develop in gregarious species. Selection for larger females is generally constrained by a trade‐off between the benefits of increased fecundity at the adult stage and the higher predation risk at the larval stage associated with longer development times (Allen et al. 2011). This constraint might be alleviated in the unpalatable larvae of *Heliconius*, as bigger larval and adult size could increase the strength of the warning toxic signal to predators (Jiggins 2016).

An extensive survey identified that only six percent of lepidopteran species exhibit male‐biased sexual size dimorphism, and that these patterns were generally explained by male‐male competition (i.e., intrasexual selection), in which larger males had a competitive advantage (Stillwell et al. 2010; Allen et al. 2011). In contrast, nearly half of the *Heliconius* species studied here have male‐biased sexual size dimorphism, and all of these lay eggs individually and have solitary larvae (Fig. 2). Male‐male competition is high for *Heliconius* species, as females rarely re‐mate despite their very long reproductive life‐spans (Merrill et al. 2015). In addition, large reproductive investments in the form of nuptial gifts from males can, in principle, explain male‐biased sexual size dimorphisms, as is the case in the polyandrous butterfly *Pieris napi* whose male spermatophore contains the amount of nitrogen equivalent to 70 eggs (Karlsson 1998; Allen et al. 2011). Male *Heliconius* spermatophores are not only nutrient‐rich, but also loaded with anti‐aphrodisiac pheromones that prevent re‐mating of fertilized females (Schulz et al. 2008; Merrill et al. 2015). Therefore, it seems likely that in species that lay eggs individually, sexual selection favoring larger males exceeds selection pressures for the large female size needed to carry multiple mature eggs. To our knowledge, *Heliconius* is the first example of a butterfly genus in which both female‐ and male‐biased size dimorphism are found and can be explained by contrasting reproductive strategies.

We found a strong phylogenetic signal for wing area, with species from the erato clade being on average 12% smaller than those in the melpomene clade (Fig. 3). There are many ecological factors that could explain this pattern, and all could have contributing effects that are hard to disentangle (Fig. 3). First, the erato clade is characterized by facultative pupal‐mating (Beltrán et al. 2007; Jiggins 2016), by which males fight for pupae, guard them, and mate with females as they are emerging from the pupal case (Deinert et al. 1994; Jiggins 2016). Smaller males have been shown to outcompete others for a spot on the female pupal case and more successfully inseminate emerging females compared to larger, less agile males (Deinert et al. 1994), which would remove the potential choice of females for larger males. Second, pupal‐mating seems to have far‐reaching impacts on species life‐histories (Boggs 1981). Species in the melpomene or adult‐mating clade are polyandrous, which leads to selection favoring large spermatophores (Boggs 1981) to provide mated females with abundant nutritional resources and defenses that prevent them from re‐mating with other males (Cardoso et al. 2009; Cardoso and Silva 2015). This could decrease selection pressure for larger males in the pupal‐mating clade, as nuptial gifts need not be so large or nutrient/defense rich, leading to smaller male and female offspring. However, the single origin of pupal‐mating in *Heliconius* (Fig. 2) makes it challenging to infer the impacts of this mating strategy on wing morphology, as the behavior is confounded by phylogeny.

Wing area across species positively correlated with altitude in the erato clade (Fig. 4B), but no clear pattern was found for the melpomene clade species here studied. In contrast, wing area variation within‐species (*H. erato* and *H. melpomene*) was more correlated with geography (distance to Equator, longitude) and allometry than with altitude (Fig. S10). Nevertheless, high‐altitude populations of *H. melpomene* were slightly bigger than their lowland conspecifics, whereas *H*. erato did not change (Fig. S13). Two major environmental factors are known to affect insect size across altitudinal clines. One is temperature, such that at lower temperatures, development times are longer and insects grow larger (Chown and Gaston 2010). This perhaps explains cases of Bergmann's rule among ectotherms, where larger species are found in colder climates (Shelomi 2012; Classen et al. 2017). In the geographical range here studied (Fig. 1), we predict temperatures to vary more dramatically along elevation gradients than latitudinal gradients (García‐Robledo et al. 2016). We found some evidence for species being bigger with increasing latitudes when accounting for phylogeny and allometry (Table S4), in accordance with Bergmann's rule, but more species at the extremes of the ranges are needed to clarify this (Fig. S7).

Wing beat frequency tends to be lower at low temperatures, so larger wings are required to compensate and gain the extra lift required for flight, as seen in *Drosophila robusta* (Azevedo et al. 1998; Dillon 2006). A second factor likely to contribute to altitude related differences in wing area is air pressure changes and the correlated lower oxygen availability, which affects flight motion and kinematics as well as many physiological processes. High‐altitude insects can minimize the impacts of lower air pressure by having larger wings, because this lowers the velocity required to induce flight (Dudley 2002).

### HERITABILITY

Our study demonstrates that multiple selective forces may be affecting *Heliconius* wing area and aspect ratio. However, this raises the question of how plastic these traits are in the wild. In *Drosophila*, the genetic architecture of wing aspect ratio appears to be complex (Gilchrist and Partridge 2001), and is independent of that of wing area (Carreira et al. 2011). Within‐species variability of wing area halved when flies were reared in controlled conditions compared to wild populations whereas wing shape variability remained the same, but both traits had a detectable and strong heritable component (Bitner‐Mathé and Klaczko 1999). In this study, we found that 74% of the variation in wing aspect ratio could be explained by species identity, in contrast to 48% of the variation in wing area. This high and moderate intraclass repeatability is indicative of heritable traits (Nakagawa and Schielzeth 2013). The fact that closely related species are more likely to have similar wing morphologies, that is, phylogenetic signal, is also indicative of species‐level heritability (Queiroz and Ashton 2004).

In insects, wing shape is functionally more constrained than wing size. For example, genetic manipulations of wing shape in *Drosophila melanogaster* have shown that even subtle changes can have huge biomechanic impacts (Ray et al. 2016), whereas wing/body size differences may impact fecundity more than survival. Here, we find size differences between sexes that can be explained by reproductive strategy, and are likely to be genetically controlled as most sexual dimorphisms are (Allen et al. 2011).The patterns of variation in size across altitudes or latitudes are often not due to phenotypic plasticity alone, as many studies have shown their retention when populations are reared in common‐garden conditions (Chown and Gaston 2010). In Monarch butterflies, for example, common‐garden reared individuals from wild populations that had different migratory habits showed a strong genetic component for both wing aspect ratio and size (Altizer and Davis 2010).

We have shown that different selection pressures may be shaping the evolution of wing morphology in *Heliconius* and that the strength of these varies across sexes and environmental clines. Interestingly some of these patterns are maintained at the intraspecific level, with high‐altitude populations of *H. erato* and *H*. melpomene having rounder wings (Fig. 5), thus potentially adapting locally to the environment in the same way that species of this genus have adapted to altitude over longer evolutionary timescales (Fig. 4). Future work should assess the adaptive significance, plasticity, and heritability of these traits with common‐garden rearing and physiological assays in controlled conditions.

## Conclusions

Here, we have demonstrated how an understanding of natural and evolutionary history can help to disentangle the putative agents of selection on an adaptive trait. Wing trait differences across sexes, clades, and environments give insight into the selective forces driving phenotypic divergence in *Heliconius*, beyond the effects of natural selection imposed by Müllerian mimicry. Our study highlights the complexity of selection pressures affecting seemingly simple traits and the need for a thorough understanding of life history differences amongst species.

Associate Editor: M. Matos

Handling Editor: T. Chapman

## Supporting information


**Table S1**. Study species summary data.
**Table S2**. Study species sexual dimorphism.
**Table S3**. Weighted PGLS model selection table for species 15 sexual size dimorphism 16 (SSD), mean wing aspect ratio and mean wing area based on AICc.
**Table S4**. Phylogenetic Generalised Least Squares full 25 model summaries for sexual 26 size dimorphism, wing shape and wing size.
**Table S5**. Model selection based on AIC of within species variation 32 in wing aspect 33 ratio and wing area of *H. erato* and *H. melpomene*.
**Table S6**. Full model output table for within‐species (39 *H. erato* and *H. melpomene*) 40 analyses of wing aspect ratio and wing area.
**Figure S1**. Number of *Heliconius* individuals in this study collected across 3‐year 48 intervals.
**Figure S2**. Wing area (mm2, A) and wing aspect ratio (wing roundness, B) variation 53 across species.
**Figure S3**. Abouheif C‐mean distribution plots for six variables.
**Figure S4**. Sexual wing aspect ratio dimorphism across species of the erato cade 69 (A) and the melpomene clade (B).
**Figure S5**. Phylogenetic signal in wing shape.
**Figure S6**. Phylogenetic signal in wing size.
**Figure S7**. Local Moran's I index values for each species for wing area mean (left) 104 and wing aspect ratio mean (right).
**Figure S8**. Wing area variation with altitude across individuals from all species of the 111 erato clade (blue) and the melpomene clade (orange).
**Figure S9**. Species variation in wing area. Plot shows the correlation between 120 distance from the Equator (degrees) and species mean wing area (mm2).
**Figure S10**. Within‐species variation in wing area (mm2) across alt.s in *H. erato* 130 (blue) and *H. melpomene* (orange), females (left) and males (right).
**Figure S11**. Species variation in raw wing aspect ratio (A) and wing area (B) in *H*. 140 *erato* (blue) and *H. melpomene* (orange).
**Figure S12**. Wing aspect ratio (A) and area (B) variation across mimicry ring wing 149 patterns of the two most abundant species, *H. erato* (blue) and *H. melpomene* 150 (orange).
**Figure S13**. Relative importance of model predictors of within species variation wing 157 aspect ratio (A, B) and wing area (C, B) in *H. erato* (A, C) and *H. melpomene* (B, D).

## References

[evo13865-bib-0092] Abouheif, E. 1999 A method for testing the assumption of phylogenetic independence in comparative data. Evol. Ecol. Res. 1:895–909.

[evo13865-bib-0001] Allen, C. E. , B. J. Zwaan , and P. M. Brakefield . 2011 Evolution of sexual dimorphism in the Lepidoptera. Annu. Rev. Entomol. 56:445–464. 20822452 10.1146/annurev-ento-120709-144828

[evo13865-bib-0002] Altizer, S. , and A. K. Davis . 2010 Populations of monarch butterflies with different migratory behaviors show divergence in wing morphology. Evolution 64:1018–1028. 20067519 10.1111/j.1558-5646.2010.00946.x

[evo13865-bib-0003] Azevedo, R. B. R. , A. C. James , J. McCabe , and L. Partridge . 1998 Latitudinal variation of wing: thorax size ratio and wing‐aspect ratio in *Drosophila melanogaster* . Evolution 52:1353–1362. 28565379 10.1111/j.1558-5646.1998.tb02017.x

[evo13865-bib-0004] Barton, K. and M. K. Barton , 2019 Package ‘MuMIn’. *Multi‐model inference. version, 1*(6).

[evo13865-bib-0005] Beltrán, M. , C. Jiggins , A. Brower , E. Bermingham , and J. Mallet . 2007 Do pollen feeding and pupal‐mating have a single origin in *Heliconius* butterflies? Inferences from multilocus sequence data. Biol. J. Linn. Soc. 92:221–239.

[evo13865-bib-0006] Berwaerts, K. , H. Van Dyck , and P. Aerts . 2002 Does flight morphology relate to flight performance? An experimental test with the butterfly *Pararge aegeria* . Funct. Ecol. 16:484–491.

[evo13865-bib-0007] Betts, C. R. , and R. J. Wootton . 1988 Wing shape and flight behaviour in butterflies (Lepidoptera: Papilionoidea and Hesperioidea): a preliminary analysis. J. Exp. Biol. 138:271–288.

[evo13865-bib-0008] Bitner‐Mathé, B. C. , and L. B. Klaczko . 1999 Size and shape heritability in natural populations of *Drosophila mediopunctata*: temporal and microgeographical variation. Heredity 105:35–42. 10.1023/a:1003591726851 10483092

[evo13865-bib-0009] Boggs, C. L. 1981 Selection pressures affecting male nutrient investment at mating in *Heliconiine* butterflies. Evolution 35:931–940. 28581061 10.1111/j.1558-5646.1981.tb04959.x

[evo13865-bib-0010] Breuker, C. J. , P. M. Brakefield , and M. Gibbs . 2007 The association between wing morphology and dispersal is sex‐specific in the glanville fritillary butterfly *Melitae cinxia* (Lepidoptera: Nymphalidae). Eur. J. Entomol. 104:445–452.

[evo13865-bib-0011] Buler, J. J. , R. J. Lyon , J. A. Smolinsky , T. J. Zenzal , and F. R. Moore . 2017 Body mass and wing shape explain variability in broad‐scale bird species distributions of migratory passerines along an ecological barrier during stopover. Oecologia 185:205–212. 28852874 10.1007/s00442-017-3936-y

[evo13865-bib-0012] Cardoso, M. Z. , J. J. Roper , and L. E. Gilbert . 2009 Prenuptial agreements: mating frequency predicts gift‐giving in Heliconius species. Entomol. Exp. Appl. 131:109–114.

[evo13865-bib-0013] Cardoso, M. Z. , and E. S. Silva . 2015 Spermatophore quality and production in two *Heliconius* butterflies with contrasting mating systems. J. Insect Behav. 28:693–703.

[evo13865-bib-0014] Carreira, V. P. , I. M. Soto , J. Mensch , and J. J. Fanara . 2011 Genetic basis of wing morphogenesis in Drosophila: sexual dimorphism and non‐allometric effects of shape variation. BMC Dev. Biol. 11:1–32. 21635778 10.1186/1471-213X-11-32 PMC3129315

[evo13865-bib-0015] Cespedes, A. , C. M. Penz , and P. J. Devries . 2015 Cruising the rain forest floor: butterfly wing shape evolution and gliding in ground effect. J. Anim. Ecol. 84:808–816. 25484251 10.1111/1365-2656.12325

[evo13865-bib-0016] Chai, P. , and R. B. Srygley . 1990 Predation and the flight, morphology, and temperature of neotropical rain‐forest butterflies. Am. Nat. 135:748–765.

[evo13865-bib-0017] Chazot, N. , S. Panara , N. Zilbermann , P. Blandin , Y. Le Poul , R. Cornette , M. Elias , and V. Debat . 2016 Morpho morphometrics: shared ancestry and selection drive the evolution of wing size and shape in Morpho butterflies. Evolution 70:181–194. 26688277 10.1111/evo.12842

[evo13865-bib-0018] Chown, S. L. , and K. J. Gaston . 2010 Body size variation in insects: a macroecological perspective. Biol. Rev. 85:139–169. 20015316 10.1111/j.1469-185X.2009.00097.x

[evo13865-bib-0019] Classen, A. , I. Steffan‐Dewenter , W. J. Kindeketa , and M. K. Peters . 2017 Integrating intraspecific variation in community ecology unifies theories on body size shifts along climatic gradients. Funct. Ecol. 31:768–777.

[evo13865-bib-0020] Cook, L. M. , E. W. Thomason , and A. M. Young . 1976 Population structure, dynamics and dispersal of the tropical butterfly *Heliconius charitonius* . J. Anim. Ecol. 45:851–863.

[evo13865-bib-0021] Deinert, E. I. , J. T. Longino , and L. E. Gilbert . 1994 Mate competition in butterflies. Nature 370:23–24.

[evo13865-bib-0022] Dell'Aglio, D. D. , M. E. Losada , and C. D. Jiggins . 2016 Butterfly learning and the diversification of plant leaf shape. Front. Ecol. Evol. 4:81.

[evo13865-bib-0023] DeVries, P. J. , C. M. Penz , and R. I. Hill . 2010 Vertical distribution, flight behaviour and evolution of wing morphology in Morpho butterflies. J. Anim. Ecol. 79:1077–1085. 20487088 10.1111/j.1365-2656.2010.01710.x

[evo13865-bib-0024] Dillon, M. E. 2006 Into thin air: Physiology and evolution of alpine insects. Integr. Comp. Biol. 46:49–61. 21672722 10.1093/icb/icj007

[evo13865-bib-0025] Dillon, M. E. , M. R. Frazier , R. Dudley , S. Integrative , C. Biology , N. Feb , M. E. Dillon , M. R. Frazier , and R. Dudleyt . 2018 Into thin air: Physiology and evolution of alpine insects stable. Integr. Compar. Biol. 46:49–61. 10.1093/icb/icj007 21672722

[evo13865-bib-0026] Dudley, R. 2002 The biomechanics of insect flight: form, function, evolution. Ann. Entomol. Soc. Am. 93:1195–1196.

[evo13865-bib-0027] Dunn, P. O. , J. K. Armenta , and L. A. Whittingham . 2015 Natural and sexual selection act on different axes of variation in avian plumage color. Sci. Adv. 1:e1400155. 26601146 10.1126/sciadv.1400155 PMC4643820

[evo13865-bib-0028] Farney, J. , and E. D. Fleharty . 1969 Aspect ratio, loading, wing span, and membrane areas of bats. J. Mammal. 50:362.

[evo13865-bib-0029] Freckleton, R. P. , P. H. Harvey , and M. Pagel . 2002 Phylogenetic analysis and comparative data: a test and review of evidence. Am. Nat. 160:712. 18707460 10.1086/343873

[evo13865-bib-0030] Garamszegi, L. Z. 2014 Modern phylogenetic comparative methods and their application in evolutionary biology. Springer, Berlin, Germany.

[evo13865-bib-0031] García‐Robledo, C. , E. K. Kuprewicz , C. L. Staines , T. L. Erwin , and W. J. Kress . 2016 Limited tolerance by insects to high temperatures across tropical elevational gradients and the implications of global warming for extinction. Proc. Natl. Acad. Sci. U. S. A. 113:680–685. 26729867 10.1073/pnas.1507681113 PMC4725502

[evo13865-bib-0032] Gilchrist, A. S. , R. B. R. Azevedo , L. Partridge , and P. O'Higgins . 2000 Adaptation and constraint in the evolution of Drosophila melanogaster wing shape. Evol. Dev. 2:114–124. 11258389 10.1046/j.1525-142x.2000.00041.x

[evo13865-bib-0033] Gilchrist, A. S. , and L. Partridge . 2001 The contrasting genetic architecture of wing size and shape in Drosophila melanogaster. Heredity 86:114–152. 10.1046/j.1365-2540.2001.00779.x 11380659

[evo13865-bib-0034] Ginestet, C. 2011 ggplot2: elegant graphics for data analysis. J. R. Stat. Soc. A 174:245–246.

[evo13865-bib-0035] Grömping, U. 2006 Relative importance for linear regression in R: the package relaimpo. J. Stat. Softw. 17 10.18637/jss.v017.i01.

[evo13865-bib-0036] Hurvich, C. M. , and C.‐L. Tsai . 1989 Regression and time series model selection in small samples. Biometrika 76:297–307.

[evo13865-bib-0037] Jiggins, C. D. 2016 The ecology and evolution of Heliconius butterflies. Oxford Univ. Press, Oxford, U.K.

[evo13865-bib-0038] Jombart, T. , and S. Dray . 2010 Adephylo: exploratory analyses for the phylogenetic comparative method. Bioinformatics 26:1907–1909. 20525823 10.1093/bioinformatics/btq292

[evo13865-bib-0039] Jones, R. T. , Y. L. Poul , A. C. Whibley , C. Mérot , R. H. ffrench‐Constant , and M. Joron . 2013 Wing shape variation associated with mimicry in butterflies. Evolution 67:2323–2334. 23888854 10.1111/evo.12114

[evo13865-bib-0040] Joron, M. 2005 Polymorphic mimicry, microhabitat use, and sex‐specific behaviour. J. Evol. Biol. 18:547–56. 15842484 10.1111/j.1420-9101.2005.00880.x

[evo13865-bib-0041] Karlsson, B. 1998 Nuptial gifts, resource budgets, and reproductive output in a polyandrous butterfly. Ecology 79:2931.

[evo13865-bib-0042] Keck, F. , F. Rimet , A. Bouchez , and A. Franc . 2016 Phylosignal: an R package to measure, test, and explore the phylogenetic signal. Ecol. Evol. 6:2774–2780. 27066252 10.1002/ece3.2051 PMC4799788

[evo13865-bib-0043] Kemp, D. J. 2019 Manipulation of natal host modifies adult reproductive behaviour in the butterfly *Heliconius charithonia* . Proc. R. Soc. B Biol. Sci. 286:20191225. 10.1098/rspb.2019.1225 PMC6742987 31506053

[evo13865-bib-0045] Klepsatel, P. , M. Gáliková , C. D. Huber , and T. Flatt . 2014 Similarities and differences in altitudinal versus latitudinal variation for morphological traits in *Drosophila melanogaster* . Evolution 68:1385–1398. 24410363 10.1111/evo.12351

[evo13865-bib-0046] Kozak, K. M. , W. O. McMillan , M. Joron , and C. D. Jiggins . 2018 Genome‐wide admixture is common across the Heliconius radiation. bioRxiv 414201. 10.1093/gbe/evab099 PMC8283734 33944917

[evo13865-bib-0047] Kozak, K. M. , N. Wahlberg , A. F. E. Neild , K. K. Dasmahapatra , J. Mallet , and C. D. Jiggins . 2015 Multilocus species trees show the recent adaptive radiation of the mimetic Heliconius butterflies. Syst. Biol. 64:505–524. 25634098 10.1093/sysbio/syv007 PMC4395847

[evo13865-bib-0048] Le Roy, C. , V. Debat , and V. Llaurens . 2019 Adaptive evolution of butterfly wing shape: from morphology to behaviour. Biol. Rev. 94:1261–1281. 30793489 10.1111/brv.12500

[evo13865-bib-0049] Losos, J. B. 2010 Adaptive radiation, ecological opportunity, and evolutionary determinism. Am. Nat., 175: 623–39. 20412015 10.1086/652433

[evo13865-bib-0050] Maia, R. , D. R. Rubenstein , and M. D. Shawkey . 2016 Selection, constraint, and the evolution of coloration in African starlings. Evolution 70:1064–1079. 27071847 10.1111/evo.12912

[evo13865-bib-0051] Marques, D. A. , J. I. Meier , and O. Seehausen . 2019 A combinatorial view on speciation and adaptive radiation. Trends Ecol. Evol. 34:531–544. 30885412 10.1016/j.tree.2019.02.008

[evo13865-bib-0052] Mendoza‐Cuenca, L. , and R. MacÍas‐Ordóñez . 2010 Female asynchrony may drive disruptive sexual selection on male mating phenotypes in a Heliconius butterfly. Behav. Ecol. 21:144–152.

[evo13865-bib-0053] Mérot, C. , Y. Le Poul , M. Théry , and M. Joron . 2016 Refining mimicry: phenotypic variation tracks the local optimum. J. Anim. Ecol. 85:1056–59. 27003742 10.1111/1365-2656.12521

[evo13865-bib-0054] Mérot, C. , J. Mavárez , A. Evin , K. K. Dasmahapatra , J. Mallet , G. Lamas , and M. Joron . 2013 Genetic differentiation without mimicry shift in a pair of hybridizing Heliconius species (Lepidoptera: Nymphalidae). Biol. J. Linn. Soc. 109:830–847.

[evo13865-bib-0055] Merrill, R. M. , K. K. Dasmahapatra , J. W. Davey , D. D. Dell'Aglio , J. J. Hanly , B. Huber , C. D. Jiggins , M. Joron , K. M. Kozak , V. Llaurens , et al. 2015 The diversification of Heliconius butterflies: what have we learned in 150 years? J. Evol. Biol. 28:1417–1438. 26079599 10.1111/jeb.12672

[evo13865-bib-0056] Merrill, R. M. , R. W. R. Wallbank , V. Bull , P. C. A. Salazar , J. Mallet , M. Stevens , and C. D. Jiggins . 2012 Disruptive ecological selection on a mating cue. Proc. R. Soc. B Biol. Sci. 279:4907–13. 10.1098/rspb.2012.1968 PMC3497240 23075843

[evo13865-bib-0057] Münkemüller, T. , S. Lavergne , B. Bzeznik , S. Dray , T. Jombart , K. Schiffers , and W. Thuiller . 2012 How to measure and test phylogenetic signal. Methods Ecol. Evol. 3:743–756.

[evo13865-bib-0058] Nadeau, N. J. , C. Pardo‐Diaz , A. Whibley , M. A. Supple , S. V. Saenko , R. W. R. Wallbank , G. C. Wu , L. Maroja , L. Ferguson , J. J. Hanly , et al. 2016 The gene cortex controls mimicry and crypsis in butterflies and moths. Nature 534:106–10. 27251285 10.1038/nature17961 PMC5094491

[evo13865-bib-0059] Nakagawa, S. , and H. Schielzeth . 2013 A general and simple method for obtaining R2 from generalized linear mixed‐effects models. Methods Ecol. Evol. 4:133–142.

[evo13865-bib-0060] Nosil, P. , R. Villoutreix , C. F. De Carvalho , T. E. Farkas , V. Soria‐Carrasco , J. L. Feder , B. J. Crespi , and Z. Gompert . 2018 Natural selection and the predictability of evolution in timema stick insects. Science 359:765–770. 29449486 10.1126/science.aap9125

[evo13865-bib-0061] Ortega Ancel, A. , R. Eastwood , D. Vogt , C. Ithier , M. Smith , R. Wood , and M. Kovač . 2017 Aerodynamic evaluation of wing shape and wing orientation in four butterfly species using numerical simulations and a low‐speed wind tunnel, and its implications for the design of flying micro‐robots. Interface Focus 7:20160087. 28163879 10.1098/rsfs.2016.0087 PMC5206606

[evo13865-bib-0062] Outomuro, D. , and F. Johansson . 2017 A potential pitfall in studies of biological shape: does size matter? J. Anim. Ecol. 86:1447–1457. 28699246 10.1111/1365-2656.12732

[evo13865-bib-0063] Paradis, E. 2012 Analysis of phylogenetics and evolution with R. 2nd ed Springer, New York, NY.

[evo13865-bib-0064] Pinheiro, J. , D. Bates , S. DebRoy , D. Sarkar , and R Development Core Team . 2007 nlme: linear and nonlinear mixed effects models. R package version 3.1‐141. Available via https://CRAN.R-project.org/package=nlme.

[evo13865-bib-0065] Pitchers, W. , J. E. Pool , and I. Dworkin . 2012 Altitudinal clinal variation in wing size and shape in African Drosophila melanogaster: one cline or many? Evolution 67:438–452. 23356616 10.1111/j.1558-5646.2012.01774.x PMC3786396

[evo13865-bib-0066] Pyrcz, T. W. , K. Sattler , D. C. Lees , G. W. Beccaloni , J. R. Ferrer‐Paris , J. Wojtusiak , and A. L. Viloria . 2004 A brachypterous butterfly? Proc. R. Soc. Lond. B Biol. Sci. 270, 10.1098/rsbl.2003.0015. PMC1698010 12952626

[evo13865-bib-0067] de Queiroz, A. , and K. G. Ashton . 2004 The phylogeny of a species‐level tendency: species heritability and possible deep origins of Bergmann's rule in tetrapods. Evolution 58:1674–1684. 15446422 10.1111/j.0014-3820.2004.tb00453.x

[evo13865-bib-0068] Ray, R. P. , T. Nakata , P. Henningsson , and R. J. Bomphrey . 2016 Enhanced flight performance by genetic manipulation of wing shape in Drosophila. Nat. Commun. 7:1–8. 10.1038/ncomms10851 PMC4773512 26926954

[evo13865-bib-0091] R Development Core Team . 2011 R: a language and environment for statistical computing. R Foundation for Statistical Computing, Vienna, Austria.

[evo13865-bib-0069] Revell, L. J. 2010 Phylogenetic signal and linear regression on species data. Methods Ecol. Evol. 1:319–329.

[evo13865-bib-0093] Ripley, M. B. 2011. R: package 'MASS.'

[evo13865-bib-0070] Rodrigues, D. , and G. R. P. Moreira . 2002 Geographical variation in larval host‐plant use by Heliconius erato (Lepidoptera: Nymphalidae) and consequences for adult life history. Braz. J. Biol. 62:321–332. 12489404 10.1590/s1519-69842002000200016

[evo13865-bib-0071] Rodrigues, D. , and G. R. P. Moreira 2004 Seasonal variation in larval host plants and consequences for *Heliconius erato* (Lepidoptera: Nymphalidae) adult body size. Austral Ecol. 29:437–445.

[evo13865-bib-0072] Rossato, D. O. , D. Boligon , R. Fornel , M. R. Kronforst , G. L. Gonçalves , and G. R. P. Moreira . 2018a Subtle variation in size and shape of the whole forewing and the red band among co‐mimics revealed by geometric morphometric analysis in Heliconius butterflies. Ecol. Evol. 8:3280–3295. 29607024 10.1002/ece3.3916 PMC5869215

[evo13865-bib-0073] Rossato, D. O. , L. A. Kaminski , C. A. Iserhard , and L. Duarte . 2018b More than colours: an eco‐evolutionary framework for wing shape diversity in butterflies. Pp. 55–84 *in* R. ffrench‐Constant, ed. 1st ed Vol. 54 Advances in insect physiology: butterfly wing patterns and mimicry. Academic Press, Cambridge, MA.

[evo13865-bib-0074] Rosser, N. , A. V. L. Freitas , B. Huertas , M. Joron , G. Lamas , C. Mérot , F. Simpson , K. R. Willmott , J. Mallet , and K. K. Dasmahapatra . 2019 Cryptic speciation associated with geographic and ecological divergence in two Amazonian Heliconius butterflies. Zool. J. Linn. Soc. 186:233–249.

[evo13865-bib-0075] Rosser, N. , K. M. Kozak , A. B. Phillimore , and J. Mallet . 2015 Extensive range overlap between heliconiine sister species: evidence for sympatric speciation in butterflies? BMC Evol. Biol. 15:125. 26123545 10.1186/s12862-015-0420-3 PMC4486711

[evo13865-bib-0076] Satterfield, D. A. , and A. K. Davis . 2014 Variation in wing characteristics of monarch butterflies during migration: earlier migrants have redder and more elongated wings. Anim. Migr. 2 10.2478/ami-2014-0001.

[evo13865-bib-0089] Schindelin, J. , I. Arganda‐Carreras , E. Frise , V. Kaynig , M. Longair , T. Pietzsch , S. Preibisch , C. Rueden , S. Saalfeld , B. Schmid , et al. 2012 Fiji: an open‐source platform for biological‐image analysis. Nat. Methods 9:676–682. 22743772 10.1038/nmeth.2019 PMC3855844

[evo13865-bib-0077] Schluter, D. 2000 The ecology of adaptive radiation. Oxford Univ. Press, Oxford, U.K.

[evo13865-bib-0078] Schulz, S. , C. Estrada , S. Yildizhan , M. Boppré , and L. E. Gilbert . 2008 An antiaphrodisiac in *Heliconius melpomene* butterflies. J. Chem. Ecol. 34:82–93. 18080165 10.1007/s10886-007-9393-z

[evo13865-bib-0079] Shelomi, M. 2012 Where are we now? Bergmann's Rule Sensu Lato in insects. Am. Nat. 180:511–519. 22976013 10.1086/667595

[evo13865-bib-0080] Singer, M. C. 1982 Sexual selection for small size in male butterflies. Am. Nat. 119:440–443.

[evo13865-bib-0081] Srygley, R. B. 1994 Locomotor mimicry in butterflies? the associations of positions of centres of mass among groups of mimetic, unprofitable prey. Philos. Trans. R. Soc. B Biol. Sci. 343 10.1098/rstb.1994.0017.

[evo13865-bib-0082] Srygley, R. B. 2004a The aerodynamic costs of warning signals in palatable mimetic butterflies and their distasteful models. Proc. R. Soc. B Biol. Sci. 271:589–594. 10.1098/rspb.2003.2627 PMC1691640 15156916

[evo13865-bib-0084] Stalker, H. D. , and H. L. Carson . 1948 An altitudinal transect of *Drosophila robusta* Sturtevant. Evolution 2:295–305. 18104587 10.1111/j.1558-5646.1948.tb02747.x

[evo13865-bib-0085] Stillwell, R. C. , W. U. Blanckenhorn , T. Teder , G. Davidowitz , and C. W. Fox . 2010 Sex differences in phenotypic plasticity affect variation in sexual size dimorphism in insects: from physiology to evolution. Annu. Rev. Entomol. 55:227–245. 19728836 10.1146/annurev-ento-112408-085500 PMC4760685

[evo13865-bib-0086] Stoffel, M. A. , S. Nakagawa , and H. Schielzeth . 2017 rptR: repeatability estimation and variance decomposition by generalized linear mixed‐effects models. Methods Ecol. Evol. 8:1639–1644.

[evo13865-bib-0090] Strauss, R. E. 1990 Patterns of Quantitative Variation in Lepidopteran wing morphology: the convergent groups heliconiinae and ithomiinae (papilionoidea: nymphalidae). Evolution 44:86–103. 28568216 10.1111/j.1558-5646.1990.tb04281.x

[evo13865-bib-0087] Thurman, T. J. , E. Brodie , E. Evans , and W. O. McMillan . 2018 Facultative pupal mating in *Heliconius erato*: implications for mate choice, female preference, and speciation. Ecol. Evol. 8:1882–1889. 29435261 10.1002/ece3.3624 PMC5792586

[evo13865-bib-0088] Wootton, R. 1992 Functional morphology of insect wings. Annu. Rev. Entomol. 37:113–140.

[evo13865-bib-0094] Zhang, Z. 2016 Variable selection with stepwise and best subset approaches. Ann. Transl. Med. 4:136. 27162786 10.21037/atm.2016.03.35 PMC4842399

