# Supplementary material for: Altitude and life‐history shape the evolution of Heliconius wings
Source: Evolution. 2019 Nov 6;73(12):2436–50. doi: 10.1111/evo.13865 (PMC6916360; doi:10.1111/evo.13865)
Supplement: Supplementary file 1 — Table S1. Study species summary data. Table S2. Study species sexual dimorphism. Table S3. Weighted PGLS model selection table for species 15 sexual size dimorphism 16 (SSD), mean wing aspect ratio and mean wing area based on AICc. Table S4. Phylogenetic Generalised Least Squares full 25 model summaries for sexual 26 size dimorphism, wing shape and wing size. Table S5. Model selection based on AIC of within species variation 32 in wing aspect 33 ratio and wing area of H. erato and H. melpomene. Table S6. Full model output table for within‐species (39 H. erato and H. melpomene) 40 analyses of wing aspect ratio and wing area. Figure S1. Number of Heliconius individuals in this study collected across 3‐year 48 intervals. Figure S2. Wing area (mm2, A) and wing aspect ratio (wing roundness, B) variation 53 across species. Figure S3. Abouheif C‐mean distribution plots for six variables. Figure S4. Sexual wing aspect ratio dimorphism across species of the erato cade 69 (A) and the melpomene clade (B). Figure S5. Phylogenetic signal in wing shape. Figure S6. Phylogenetic signal in wing size. Figure S7. Local Moran's I index values for each species for wing area mean (left) 104 and wing aspect ratio mean (right). Figure S8. Wing area variation with altitude across individuals from all species of the 111 erato clade (blue) and the melpomene clade (orange). Figure S9. Species variation in wing area. Plot shows the correlation between 120 distance from the Equator (degrees) and species mean wing area (mm2). Figure S10. Within‐species variation in wing area (mm2) across alt.s in H. erato 130 (blue) and H. melpomene (orange), females (left) and males (right). Figure S11. Species variation in raw wing aspect ratio (A) and wing area (B) in H. 140 erato (blue) and H. melpomene (orange). Figure S12. Wing aspect ratio (A) and area (B) variation across mimicry ring wing 149 patterns of the two most abundant species, H. erato (blue) and H. melpomene 150 (orange). Figure S13. Relative im [file EVO-73-2436-s001.pdf]

Supplementary Materials

Table S1 Study species summary data. Sample sizes (N) and wing parameters for the 13 study species, ordered phylogenetically based on the most recent *Heliconius* phylogeny (Kozak et al. 2015). Male ratio refers to proportion of males in the sample.

Species	N	Area mean (mm^2)	Area S.E.	Aspect ratio mean	Aspect ratio S.E.	Alt. mean (m.a.s.l.)	N_{male}	N_{female}	Male ratio
H. telesiphe	48	519.4	8.9	2.35	0.009	1302	40	8	0.83
H. clysonymus	57	537.3	8.4	2.31	0.012	1346	40	17	0.70
H. erato	1687	465.8	1.5	2.09	0.002	700	1202	447	0.73
H. eleuchia	102	500.6	8.6	2.03	0.007	1408	72	30	0.71
H. sara	225	387.2	2.9	2.17	0.006	420	164	61	0.73
H. xanthocles	36	514.6	10.3	2.04	0.009	1044	20	8	0.71
H. hierax	37	512.1	8.3	2.08	0.008	1364	29	8	0.78
H. doris	42	547.5	7.1	2.30	0.012	444	34	7	0.83
H. timareta	195	606.7	3.1	2.05	0.004	883	163	32	0.84
H. cydno	127	575.1	5.5	2.09	0.007	844	112	15	0.88
H. melpomene	867	533.3	1.9	2.05	0.002	789	683	159	0.81
H. numata	44	611.6	12.9	2.11	0.013	561	30	14	0.68
H. wallacei	48	526.3	8.2	2.18	0.011	290	37	11	0.77

Table S2. Study species sexual dimorphism. Sexual size dimorphism (SSD) and sexual shape dimorphism (SShD) two-sample t-tests summary statistics. Positive t-values indicate smaller or longer-winged (higher aspect ratio) males (Fig. 2, main text).

Species	SSD t-value	SSD d.f.	SSD p-value	SShD t-value	SShD d.f.	SShD p-value
H. telesiphe	2.57	10	<0.05*	-0.5	10	ns
H. clysonymus	1.98	24	0.06•	-1.5	39	ns
H. erato	3.30	802	<0.001***	10.4	843	<0.001***
H. eleuchia	-2.61	61	<0.01**	-2.3	48	<0.05*
H. sara	-2.45	108	<0.05*	-0.6	100	ns
H. xanthocles	-0.08	13	ns	0.5	13	ns
H. hierax	-0.50	8	ns	0.5	16	ns
H. doris	-1.92	9	0.08 •	1.4	11	ns
H. timareta	2.03	49	0.05•	-0.2	46	ns
H. cydno	0.57	18	ns	0.1	16	ns
H. melpomene	5.54	240	<0.001***	1.6	230	ns
H. numata	2.57	24	<0.05*	-0.9	33	ns
H. wallacei	-1.31	16	ns	2.2	19	<0.05*

Table S3. Weighted PGLS model selection table for species sexual size dimorphism (SSD), mean wing aspect ratio and mean wing area based on AICc. All models have the species phylogeny as correlation structure and are weighted for mean trait/fixed effects variance and sample size.

Size sexual dimorphism (SSD)

Minimal model	sisd.raw ~ 1
Maximal model	sisd.raw ~ larva + shape.mean + shsd.raw + size.mean + clade
Final model	sisd.raw ~ larva + shsd.raw + shape.mean

Step	Df	Resid. Dev.	AICc
Initial model	6	73.7	109.8
-clade	7	73.46	99.5
-size.mean	8	75.6	93.7

Wing area (size)

Minimal model	area.mean ~ 1
Maximal model	area.mean ~ shape.mean * sex.ratio + alt.mean * dist.Eq.
Final model	area.mean ~ sex.ratio + alt.mean + dist.Eq + alt.mean*lat.mean

Step	Df	Resid. Dev.	AICc
Initial model	6	129.5	165.9
-shape.mean*sex.ratio	6	126.0	162.4
-shape.mean	7	126.0	152.0

Wing aspect ratio (shape)

Minimal model	shape.mean ~ 1
Maximal model	shape.mean ~ size.mean * sex.ratio + alt.mean * dist.Eq
Final model	shape.mean ~ sex.ratio + alt.mean + dist.Eq

Step	Df	Resid. Dev.	AICc
Initial model	5	-20.4	31.6
alt.mean*lat.mean	6	-19.3	17.1
- alt.mean*size.mean	7	-17.8	8.2
- size.mean	8	-17.3	1.3

Table S4. Phylogenetic Generalised Least Squares full model summaries for sexual size dimorphism, wing shape and wing size. Correlation structures of the models are shown in the third column. Dist. Eq.= distance from Equator, SD= sexual dimorphism.

Response variable (wing trait)	Model type	Corr. structure	Fixed effects	Estimate	SE	t-value	p-value	d.f. (d.f. res.)
Size Sexual Dimorphism	PGLS (nmle)	Phylogeny, sample size	(Intercept)	-31.8	21.4	-1.5	0.08	13 (9)
			Solitary larvae	-15.8	3.0	-5.3	0.0004***	
			Shape sex dim.	1.8	1.3	1.5	0.2	
			Shape mean	17.8	10.3	1.8	0.1	
Aspect ratio	PGLS (nmle)	Phylogeny, intra-sp variance, sample size	(Intercept)	1.93	0.23	8.36	0.00	13 (9)
			Altitude	-1.5E-4	6.3E-5	-2.4	0.04*	
			Dist. Eq	-4.6E-2	1.7E-2	-2.7	0.02*	
			Sex ratio	0.70	0.28	2.5	0.03*	
Area	PGLS (nmle)	Phylogeny, intra-sp variance, sample size	(Intercept)	474.52	75.37	6.30	0.00	13 (8)
			Altitude	0.16	0.04	3.47	0.008**	
			Sex ratio	-161.75	73.97	-2.19	0.06	
			Dist. Eq.	77.21	16.91	4.57	0.002**	
			Altitude* Dist. Eq	-0.07	0.02	-4.66	0.002**	

Table S5. Model selection based on AIC of within species variation in wing aspect ratio and wing area of *H. erato* and *H. melpomene*.

A) Aspect ratio, *H. erato*

Minimal model	aspect.ratio ~ 1
Maximal model	aspect.ratio ~ area.mm2 * altitude + dist.Eq. + longitude + sex
Final model	aspect.ratio ~ area.mm2 * altitude + longitude + sex

Step	Res. Df	Res. Dev.	AIC
Initial model	1294	4.92	-7246
- dist.Eq.	1295	4.92	-7248

B) Aspect ratio, *H. melpomene*

Minimal model	aspect.ratio ~ 1
Maximal model	aspect.ratio ~ area.mm2 * altitude + dist.Eq. + longitude + sex
Final model	aspect.ratio ~ area.mm2 * altitude + longitude + sex

Step	Res. Df	Res. Dev.	AIC
Initial model	704	2.3	-4070
- dist.Eq.	705	2.3	-4072

C) Wing area, *H. erato*

Minimal model	area ~ 1
Maximal model	area ~ aspect.ratio * altitude + dist.Eq + longitude + sex
Final model	area ~ aspect.ratio * altitude + dist.Eq + longitude + sex

Step	Res. Df	Res. Dev.	AIC
Initial model	1294	4841609	10720

D) Wing area, *H. melpomene*

Minimal model	area ~ 1
Maximal model	area ~ aspect.ratio * altitude + dist.Eq + longitude + sex
Final model	area ~ aspect.ratio * altitude + dist.Eq + longitude + sex

Step	Res. Df	Res. Dev.	AIC
Initial model	704	2092210	5701

Table S6. Full model output table for within-species (*H. erato* and *H. melpomene*) analyses of wing aspect ratio and wing area. Relative R^2 per fixed effect estimated with the package *relaimpo* (Grömping 2006) and the lmg statistic.

**Trait (response)**	**Sp.**	**Fixed effects**	**Res. d.f.**	**Estimate**	**SE**	**t-value**	**p-value**	**Rel. R^2**
Aspect ratio	*H. era.*	(intercept)	1295	2.2	0.03	75.57	0.00	
		altitude		-1.6E-04	0.00	-6.50	0.00***	0.43
		sex(female)		-3.4E-02	0.01	-2.87	0.004**	0.37
		area		-1.8E-04	0.00	-3.73	0.00***	0.14
		longitude		4.9E-04	0.00	2.00	0.05	0.05
		area*alt.		2.9E-07	0.00	5.30	0.00***	0.02
	H. melp.	(intercept)	705	2.3E+00	4.2E-02	55.08	0.00	
		altitude		-1.4E-04	3.4E-05	-4.17	0.00***	0.50
		area		-3.4E-04	6.1E-05	-5.63	0.00***	0.23
		area*alt.		2.2E-07	6.4E-08	3.40	0.001**	0.10
		longitude		6.6E-04	2.6E-04	2.53	0.012*	0.09
		sex(female)		-4.0E-02	1.3E-02	-3.16	0.001**	0.08
		sex(male)		-3.1E-02	1.2E-02	-2.65	0.008**	(0.08)
Wing area	*H. era.*	(intercept)	1294	879	102.27	8.60	0.00	
		AR*alt.		0.25	0.05	4.80	0.00***	0.38
		longitude		0.87	0.25	3.54	0.00***	0.18
		sex(female)		-19.3	11.90	-1.62	0.1	0.16
		sex(male)		-6.76	11.64	-0.58	0.56	0.15
		dist.Eq.		-2.35	0.84	-2.80	0.005**	(0.15)
		altitude		-0.53	0.11	-4.88	0.00***	0.08
		aspect.ratio		-161	47.56	-3.40	0.00***	0.05
	H. melp.	(intercept)	704	1430	131.35	10.89	0.00	
		dist.Eq.		-5.51	0.86	-6.38	0.00***	0.33
		longitude		1.51	0.24	6.21	0.00***	0.18
		sex(female)		-39.5	12.13	-3.26	0.001**	0.18
		sex(male)		-13.8	11.39	-1.21	0.23	(0.18)
		aspect.ratio		-365	61.86	-5.91	0.00***	0.15
		AR*alt.		0.25	0.06	3.95	0.00***	0.10
		altitude		-0.51	0.13	-3.92	0.00***	0.05

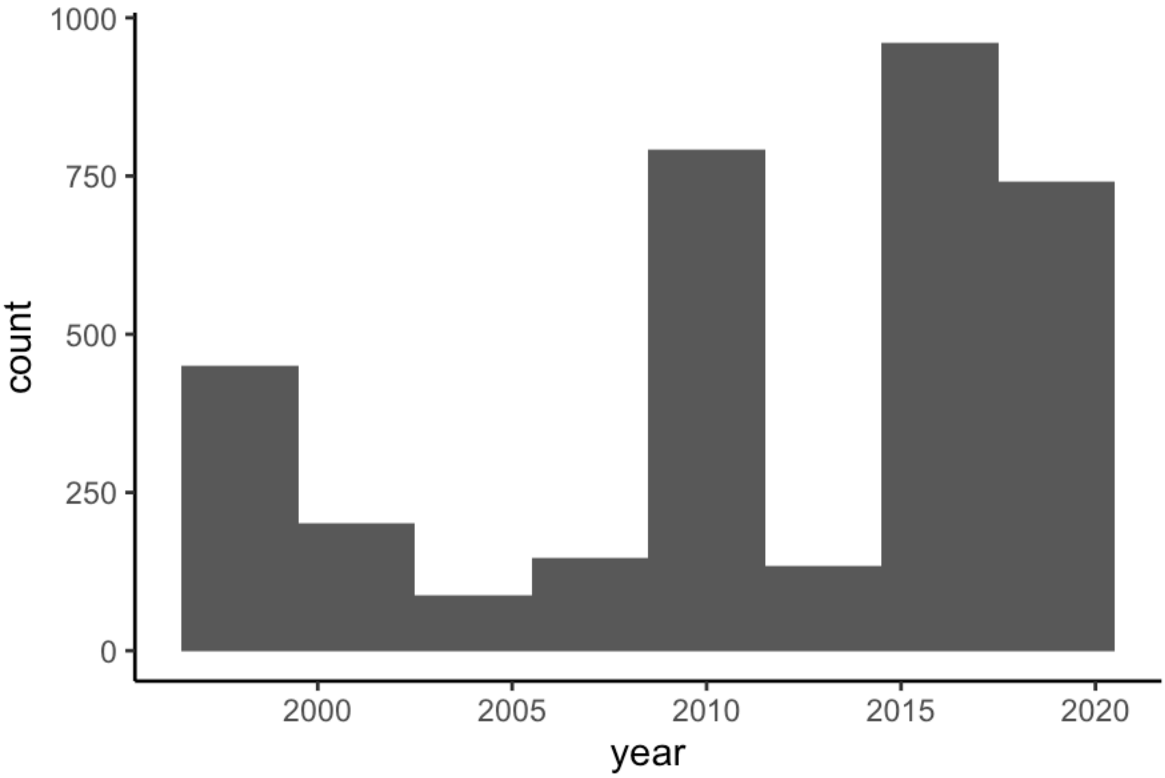

Figure S1. Number of *Heliconius* individuals in this study collected across 3-year intervals.

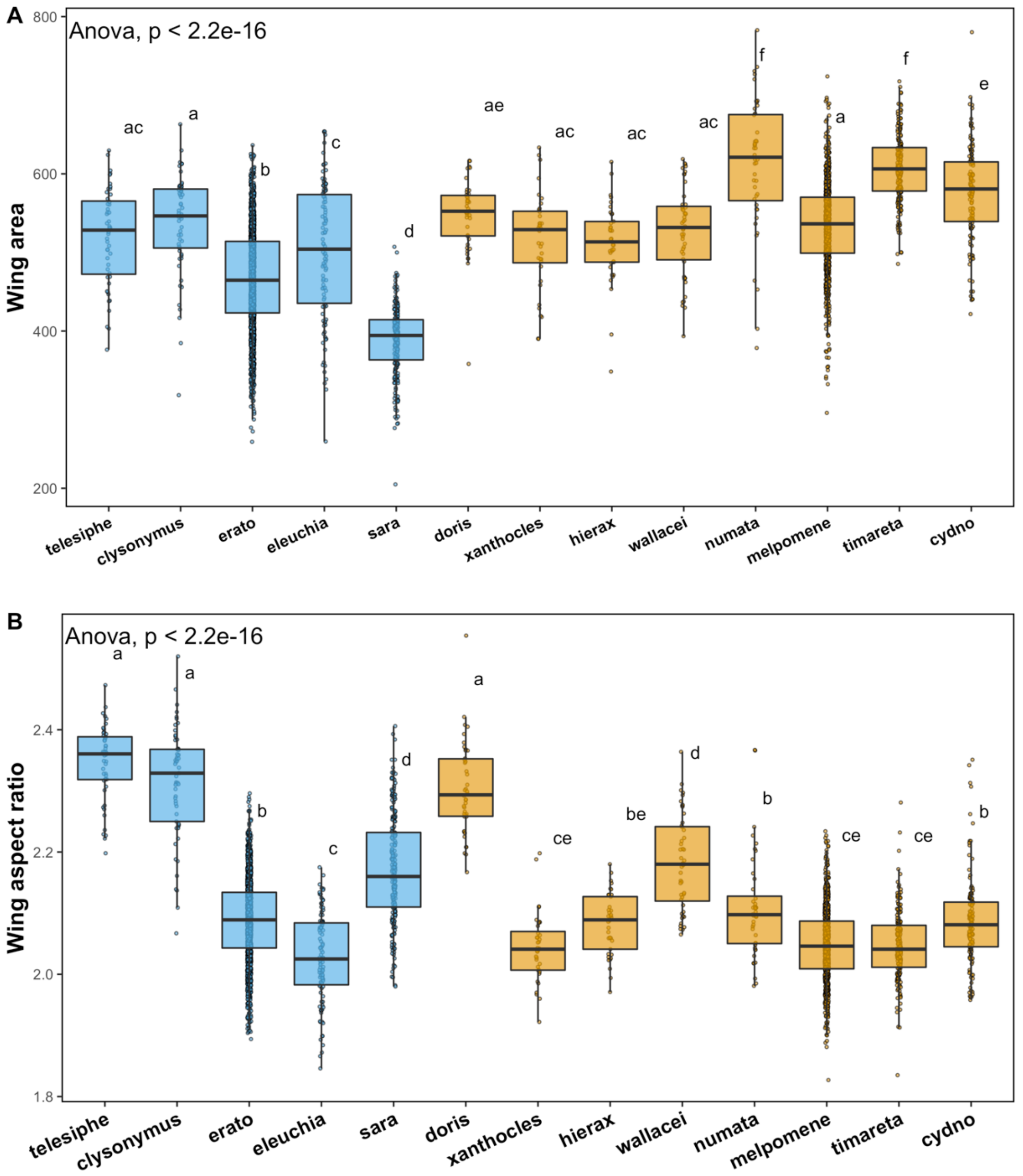

Figure S2. Wing area (mm^2, A) and wing aspect ratio (wing roundness, B) variation across species. Species sharing a letter are not significantly different (Tukey-adjusted comparisons). Species are ordered phylogenetically (for phylogeny see Fig. 3) and coloured by the two major clades.

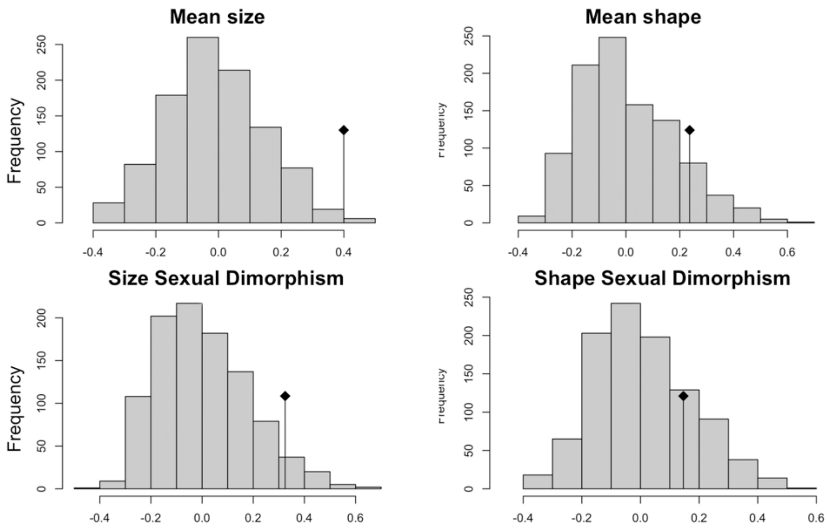

Figure S3. Abouheif C-mean distribution plots for six variables. Black dots depicts the observed C-mean statistic relative to the null hypothesis of randomisations along the tips of the phylogeny.

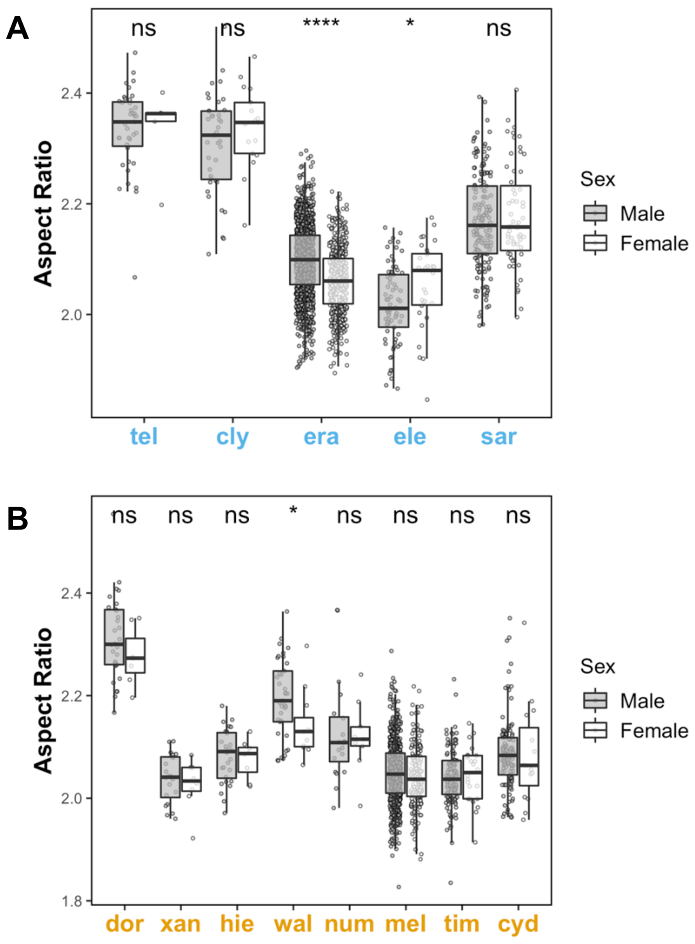

Figure S4. Sexual wing aspect ratio dimorphism across species of the erato cade (A) and the melpomene clade (B). Wing aspect ratio differences between males (grey) and females (white). Error bars represent 95% confidence intervals of the means. Stars represent significance levels of two sample t-tests between female and male wing areas for each species (•<0.08, *< 0.05, **<0.01, ***<0.001), for full t-tests output see Table S2.

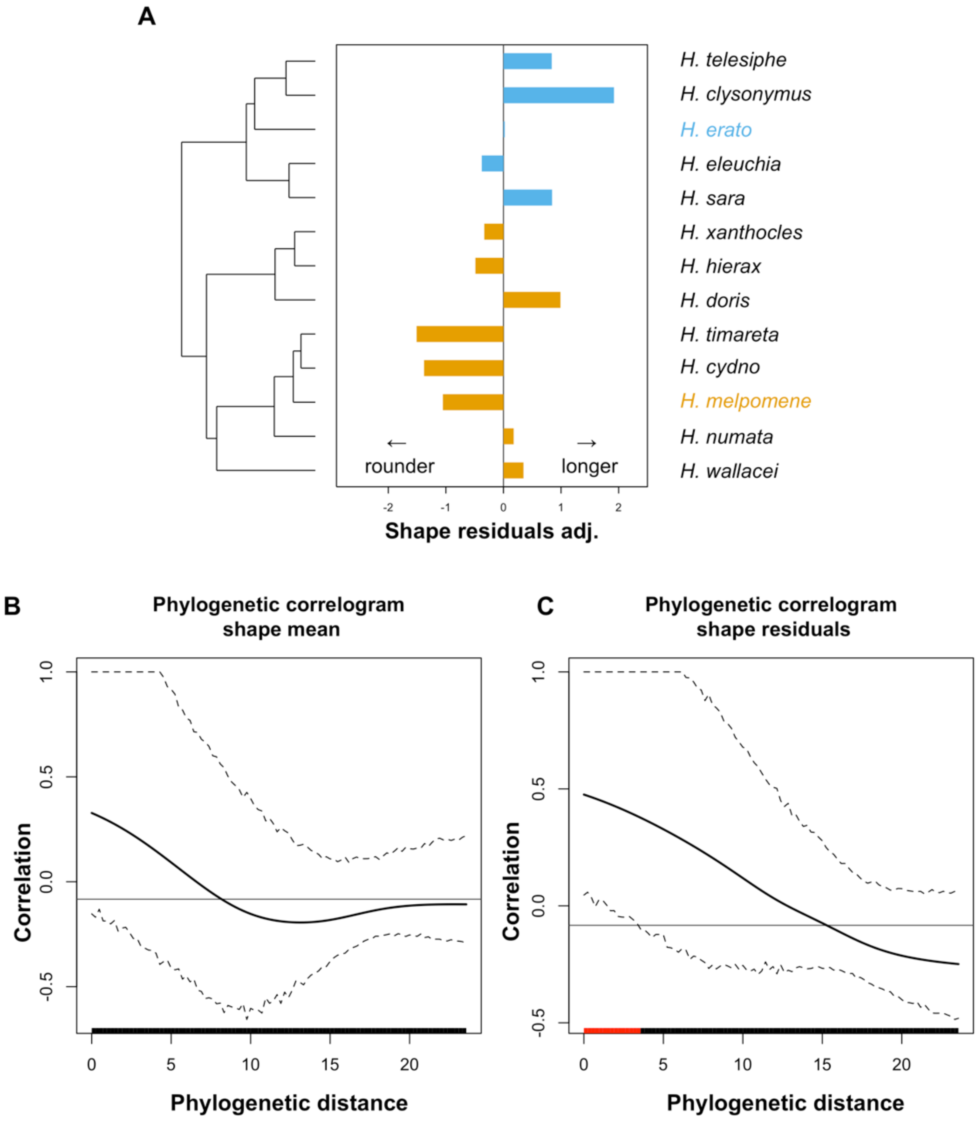

Figure S5. Phylogenetic signal in wing shape. A) Z-transformed wing shape residuals across the *Heliconius* phylogeny. B) phylogenetic correlogram of species mean wing shape. C) phylogenetic correlogram of species wing shape model residuals. The solid black line represents Moran's I index of autocorrelation and the dashed black lines represent the lower and upper bounds of the confidence 95% confidence interval. The horizontal black line represents the expected value of Moran's I under the null hypothesis of no phylogenetic autocorrelation. The coloured bars in the x-axes show whether the autocorrelation is significant (based on the confidence interval): red for significant positive autocorrelation and black for nonsignificant autocorrelation. All figures were obtained with the package phylosignal (Keck et al. 2016).

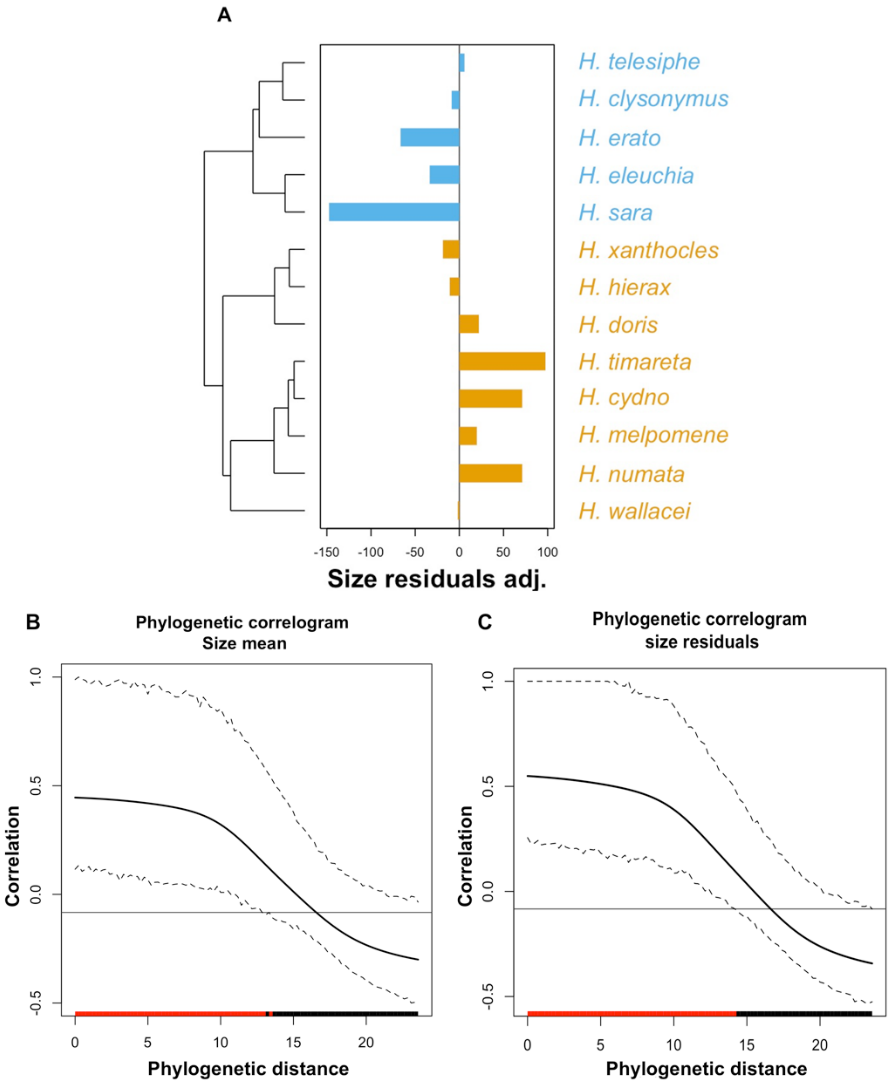

Figure S6. Phylogenetic signal in wing size. A) Centered wing size residuals across the *Heliconius* phylogeny. B) phylogenetic correlogram of species mean wing size. C) phylogenetic correlogram of species wing size model residuals. The solid black line represents Moran's I index of autocorrelation and the dashed black lines represent the lower and upper bounds of the confidence 95% confidence interval. The horizontal black line represents the expected value of Moran's I under the null hypothesis of no phylogenetic autocorrelation. The coloured bars in the x-axes show whether the autocorrelation is significant (based on the confidence interval): red for significant positive autocorrelation and black for nonsignificant autocorrelation.

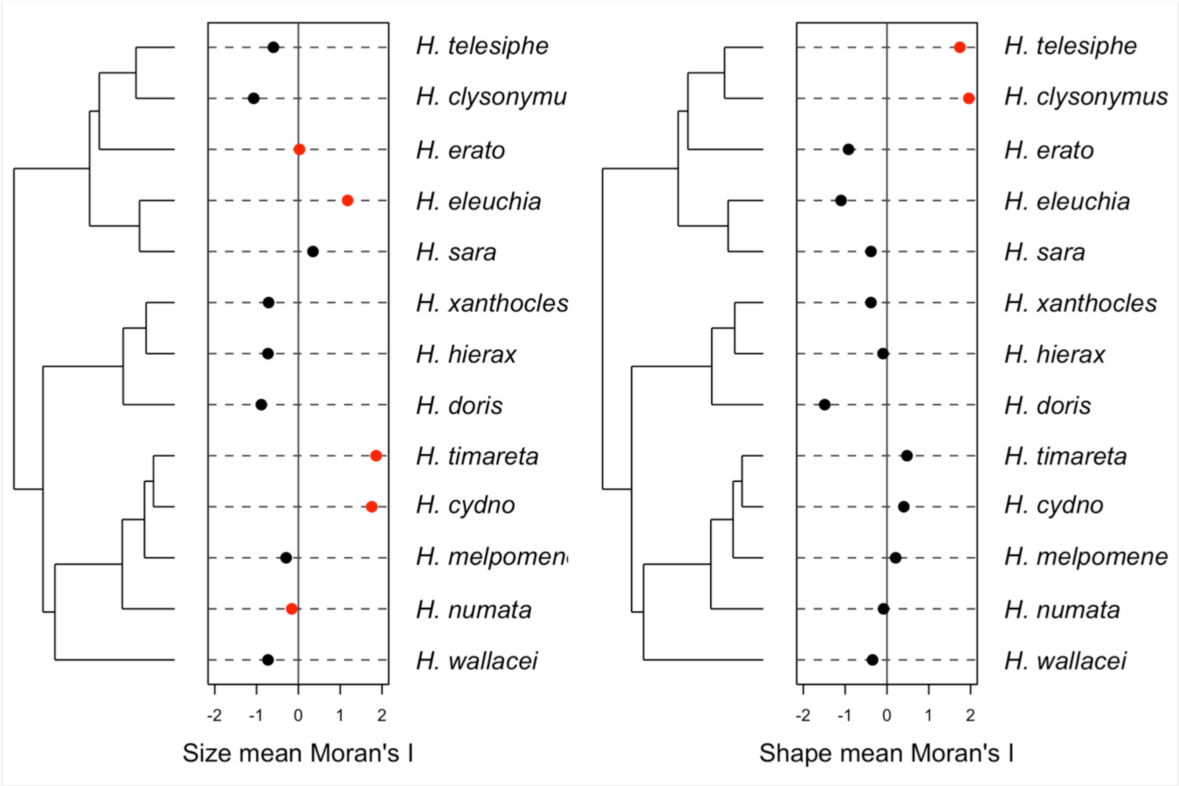

Figure S7. Local Moran's I index values for each species for wing area mean (left) and wing aspect ratio mean (right). Red points indicate significant positive autocorrelation in mean traits among neighbours in the phylogeny. Estimated and plotted with the package *phylosignal* (Keck et al. 2016).

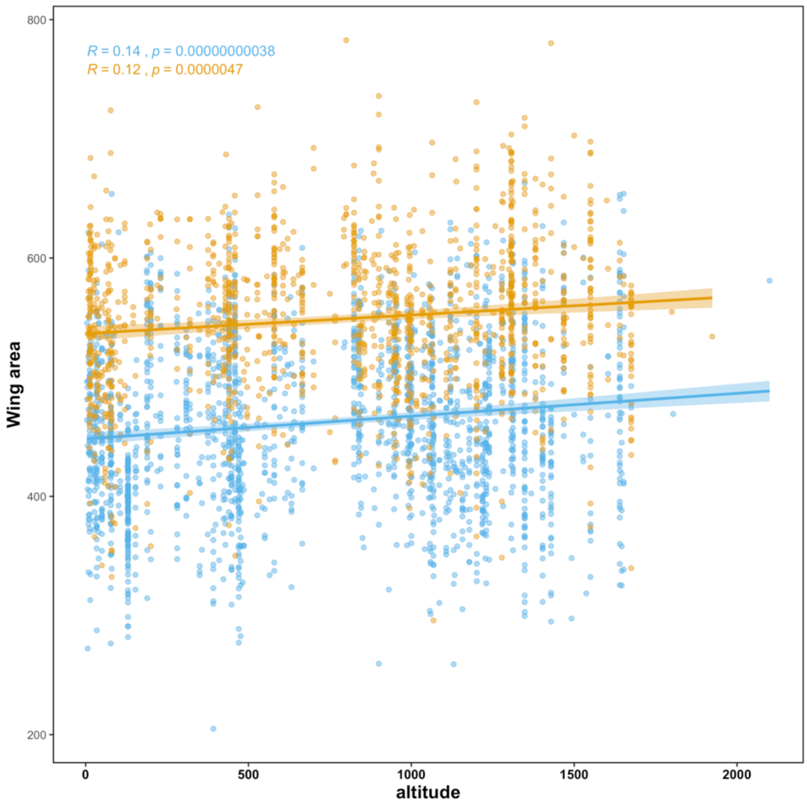

Figure S8. Wing area variation with altitude across individuals from all species of the erato clade (blue) and the melpomene clade (orange). Each point represents an individual. Lines show best linear fit and are colored by clade. Shaded areas show confidence bands at 1 standard error. Pearson correlation coefficients and p-values are shown for each regression plotted.

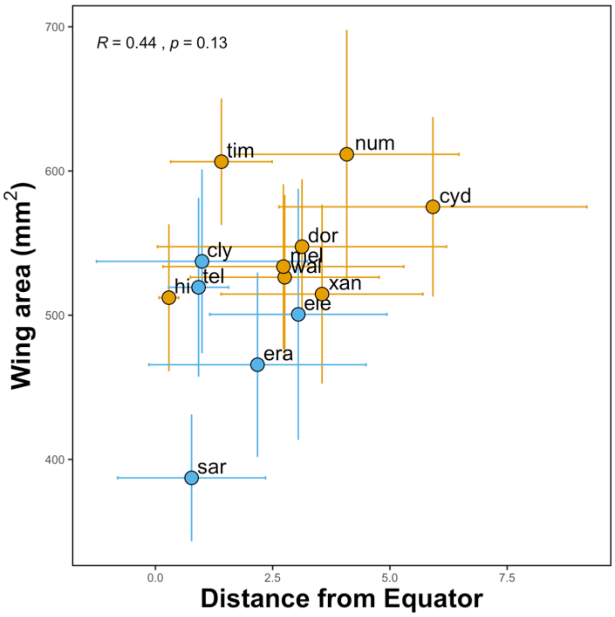

Figure S9. Species variation in wing area. Plot shows the correlation between distance from the Equator (degrees) and species mean wing area (mm2). Points represent species mean raw values per species. Horizontal and vertical lines show standard error for species mean distance from Equator and mean wing area, respectively. The point labels correspond to the first three characters of the following *Heliconius* species: *H. telesiphe*, *H. clysonymus*, *H. erato*, *H. eleuchia*, *H. sara*, *H. doris*, *H. xanthocles*, *H. hierax*, am*H. wallacei, H. numata*, *H. melpomene*, *H. timareta*, *H. cydno.*

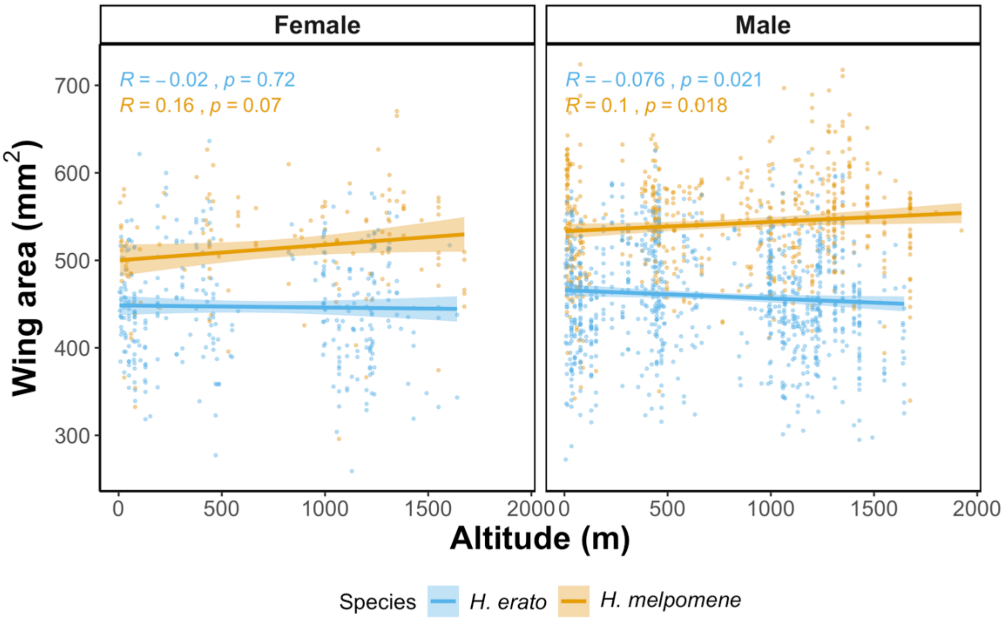

Figure S10. Within-species variation in wing area (mm^2) across alt.s in *H. erato* (blue) and *H. melpomene* (orange), females (left) and males (right). Lines show best linear fit and are colored by species. Shaded areas show confidence bands at 1 standard error. Pearson correlation coefficients and p-values are shown for each regression plotted.

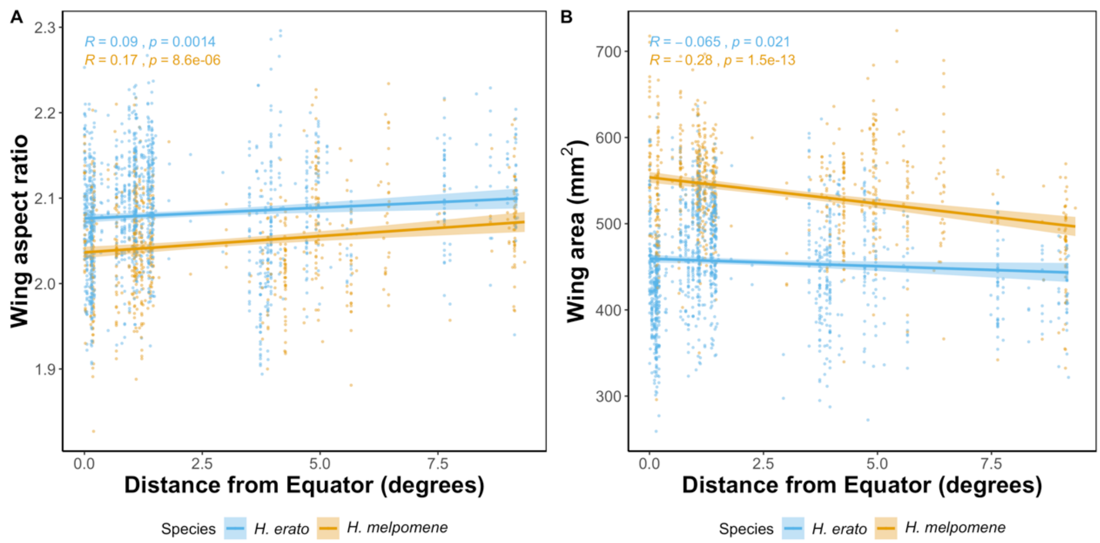

Figure S11. Species variation in raw wing aspect ratio (A) and wing area (B) in *H. erato* (blue) and *H. melpomene* (orange). Points represent individual values. Lines show best linear fit for significant effects. Shaded areas show confidence bands at 1 standard error. Pearson correlation coefficients and p-values are shown for each regression plotted.

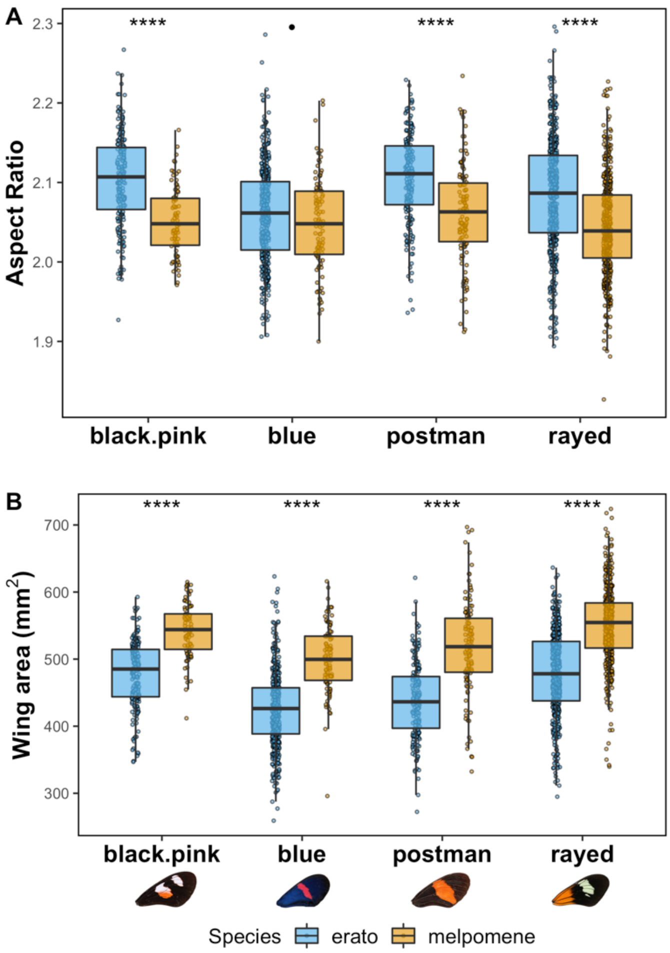

Figure S12. Wing aspect ratio (A) and area (B) variation across mimicry ring wing patterns of the two most abundant species, *H. erato* (blue) and *H. melpomene* (orange). Error bars represent 95% confidence intervals of the means. Stars represent significance levels of two sample t-tests between *H. erato* and *H. melpomene* wings for each mimicry ring (•<0.08, *< 0.05, **<0.01, ***<0.001)

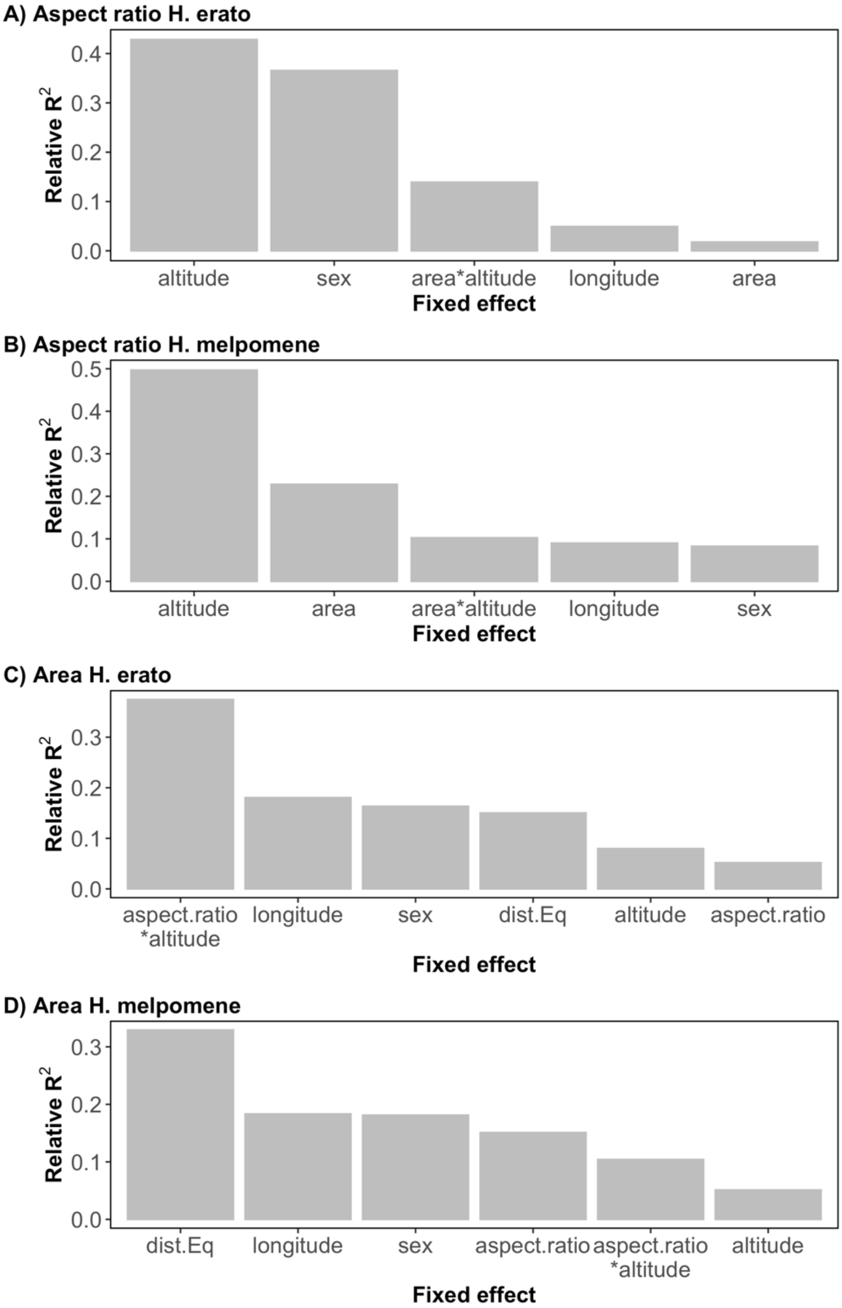

Figure S13. Relative importance of model predictors of within species variation wing aspect ratio (A, B) and wing area (C, B) in *H. erato* (A, C) and *H. melpomene* (B, D). Total model adjusted R^2 values are A) 0.13, B) 0.14, C) 0.19, D) 0.19.

References

Grömping, U. 2006. Relative Importance for Linear Regression in *R*: The Package **relaimpo**. Journal of Statistical Software, doi: 10.18637/jss.v017.i01.

Keck, F., F. Rimet, A. Bouchez, and A. Franc. 2016. Phylosignal: An R package to measure, test, and explore the phylogenetic signal. Ecology and Evolution, doi: 10.1002/ece3.2051.

Kozak, K. M., N. Wahlberg, A. F. E. Neild, K. K. Dasmahapatra, J. Mallet, and C. D. Jiggins. 2015. Multilocus species trees show the recent adaptive radiation of the mimetic heliconius butterflies. Systematic Biology 64:505–524.
